# Genetic connectivity of wolverines in western North America

**DOI:** 10.1038/s41598-024-77956-9

**Published:** 2024-11-15

**Authors:** Casey C. Day, Erin L. Landguth, Michael A. Sawaya, Anthony P Clevenger, Robert A. Long, Zachary A. Holden, Jocelyn R. Akins, Robert B. Anderson, Keith B. Aubry, Mirjam Barrueto, Nichole L. Bjornlie, Jeffrey P. Copeland, Jason T. Fisher, Anne Forshner, Justin A. Gude, Doris Hausleitner, Nichole A. Heim, Kimberly S. Heinemeyer, Anne Hubbs, Robert M. Inman, Scott Jackson, Michael Jokinen, Nathan P. Kluge, Andrea Kortello, Deborah L. Lacroix, Luke Lamar, Lisa I. Larson, Jeffrey C. Lewis, Dave Lockman, Michael K. Lucid, Paula MacKay, Audrey J. Magoun, Michelle L. McLellan, Katie M. Moriarty, Cory E. Mosby, Garth Mowat, Clifford G. Nietvelt, David Paetkau, Eric C. Palm, Kylie J.S. Paul, Kristine L. Pilgrim, Catherine M. Raley, Michael K. Schwartz, Matthew A. Scrafford, John R. Squires, Zachary J. Walker, John S. Waller, Richard D. Weir, Katherine A. Zeller

**Affiliations:** 1 University of Montana, 32 Campus Drive, Missoula, MT 59812 USA; 2 Sinopah Wildlife Research Associates, Missoula, MT USA; 3 MSU Western Transportation Institute, Bozeman, MT USA; 4 Woodland Park Zoo, Phinney Ridge, WA USA; 5 USDA Forest Service, Missoula, WA USA; 6 Cascades Carnivore Project, Hood River, OR USA; 7 Alberta Conservation Association, Crowsnest Pass, Blairmore, AB Canada; 8 University of Calgary, Alberta, CA Canada; 9 Fish & Wildlife Service, US, USA; 10 Idaho Department of Fish and Game, Boise, ID USA; 11 School of Environmental Studies, University of Victoria, Victoria, BC Canada; 12 Parks Canada, Lake Louise, AB Canada; 13 Montana Fish, Wildlife, and Parks, Helena, MT USA; 14 Selkirk College, Castlegar, BC Canada; 15 Alberta Environment and Parks, Alberta, AB Canada; 16 Braided Knowledge Consulting, Bozeman, MT USA; 17 Government of Alberta, Edmonton, AB Canada; 18 Wildlife Conservation Society, Newyork, NY USA; 19 Poisson Consulting, Nelson, Canada; 20 Simon Fraser University, Burnaby, BC Canada; 21 Swan Valley Connections, Condon, MT USA; 22 Washington Department of Fish and Wildlife, Olympia, WA USA; 23 Wyoming Game & Fish Department, Cheyenne, WY USA; 24 Alaska Department of Fish and Game, Juneau, AK USA; 25 Biodiversity Pathways, Wildlife Science Center, Kelowna, BC Canada; 26 National Council for Air and Stream Improvement, Corvallis, OR USA; 27 Ministry of Forests, British Columbia, BC Canada; 28 Wildlife Genetics International, Nelson, BC Canada; 29 Center for Large Landscape Conservation, Bozeman, MT USA; 30 Wildlife Conservation Society, Toronto, ON Canada; 31 National Park Service, West Glacier, MT USA; 32 Ministry of Water, Land and Resource Stewardship, Victoria, BC Canada

**Keywords:** Ecology, Genetics, Zoology

## Abstract

Wolverine distribution contracted along the southern periphery of its range in North America during the 19th and 20th centuries due primarily to human influences. This history, along with low densities, sensitivity to climate change, and concerns about connectivity among fragmented habitats spurred the recent US federal listing of threatened status and special concern status in Canada. To help inform large scale landscape connectivity, we collected 882 genetic samples genotyped at 19 microsatellite loci. We employed multiple statistical models to assess the landscape factors (terrain complexity, human disturbance, forest configuration, and climate) associated with wolverine genetic connectivity across 2.2 million km^2 ^of southwestern Canada and the northwestern contiguous United States. Genetic similarity (positive spatial autocorrelation) of wolverines was detected up to 555 km and a high-to-low gradient of genetic diversity occurred from north-to-south. Landscape genetics analyses confirmed that wolverine genetic connectivity has been negatively influenced by human disturbance at broad scales and positively influenced by forest cover and snow persistence at fine- and broad–scales, respectively. This information applied across large landscapes can be used to guide management actions with the goal of maintaining or restoring population connectivity.

**Supplementary Information:**

The online version contains supplementary material available at 10.1038/s41598-024-77956-9.

## Introduction

The wolverine (*Gulo gulo*) is the largest terrestrial member of the weasel family (*Mustelidae*) and is found in remote areas of the subarctic, alpine tundra, and boreal forests across North America, Europe, and Asia^1^. In North America, wolverines occur throughout Alaska and northwestern Canada, and as a small metapopulation occupying island-like, high-elevation montane regions of the northwestern United States. They naturally occur at low densities and were extirpated from their southern range in North America by about 1920^2^. Wolverines have since recolonized portions of their historic range in the contiguous United States and Canada, inhabiting the Central Rocky Mountains in the mid-20th century^3^, whereas their more recent re-colonization into the Cascade Range in BC, Canada and Washington, USA is ongoing^4^. While wolverines are now widely distributed across suitable habitat in the coterminous northwestern United States^5^, densities are low and areas with suitable conditions are fragmented. Single individuals are also occasionally found in isolated mountain ranges in California, Utah, and Colorado^6–8^. In 2013, wolverines were proposed for listing as threatened by the US Fish and Wildlife Service under the Endangered Species Act due to habitat and range loss from climate warming, harvest, and small population sizes, but listing was found not warranted due to lack of information^9^. Subsequently, through a court-mandated reevaluation of this decision, wolverines in the contiguous US were listed as threatened^10^, while in 2014 wolverines were assessed as a species of special concern in Canada^11^.

Despite their Holarctic distribution, wolverines may be experiencing population declines in some places, likely due to habitat loss in forested and montane ecosystems^12^. Furthermore, wolverines are a snow-adapted species, and models of species distribution, habitat selection, and landscape connectivity suggest that they may rely on cold microclimates and snowpack for niche space, predator avoidance, and denning, particularly at the southern extent of their range^4,13–18^. Regional habitat suitability analyses for wolverines have also identified terrain complexity, previous persecution, prey availability, and lack of human disturbance (built-up environments, human density, transportation and energy infrastructure, recreation, etc.) as potential predictors of wolverine habitat use^19–26^.

**Fig. 1:**
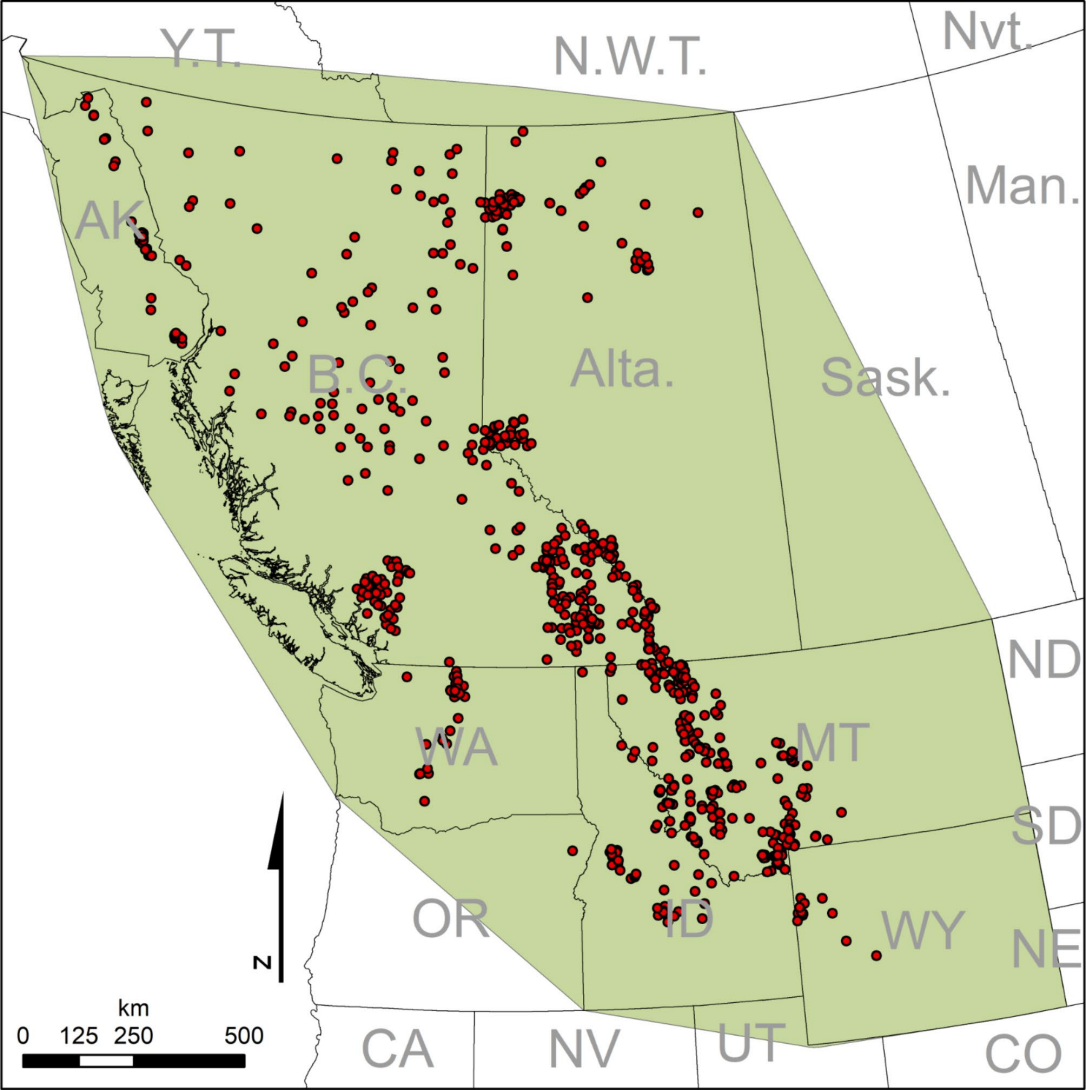
Study area. Extent of landscape connectivity analysis (green polygon) and locations of genetic samples (red points) for landscape genetics analysis of wolverines in North America.

A major management goal is to maintain connectivity among wolverine populations across large spatial extents to preserve genetic diversity and buffer against local extirpation^10,17,27^. An understanding of how landscape configuration and composition affects wolverine genetic connectivity can ultimately inform land-use planning efforts. Here, we used the largest wolverine genetic dataset ever assembled in North America, including samples from over 800 individuals collected across ~ 2.2 million km^2^ (Fig. 1), to develop models of genetic connectivity that identify landscape characteristics that promote or impede gene flow across the study area. We mapped the resulting models of landscape connectivity across a matrix of topographic, vegetative, climatic, and anthropogenic landscape variables (Table 1). We fit multiple models at variable scales and expanded on existing landscape genetics frameworks by employing validation techniques that included random and spatial cross-validation as well as individual-based simulation modeling. Specifically, we tested support for our hypothesis that landscape features explain more variation in genetic (dis)similarity than the null model of geographic distance. For the most supported model, we produced a continuous genetic connectivity surface for wolverines as a function of landscape characteristics that promote or impede genetic connectivity in western Canada and the United States. Mapped connectivity surfaces in our study area help inform ongoing efforts to identify and prioritize land preservation (e.g., protected vs. non-protected areas) and mitigation (e.g., for highway crossings or forest rehabilitation), particularly in those areas that promote genetic connectivity for populations of wolverines.

**Table 1:** Landscape variables. A summary of all variables by category rescaled to 1 km^2^ and used in the prediction of wolverine genetic connectivity. Because of correlation among variables in the same categories (*r* > 0.3), composite climate and human disturbance variables were also created using the indicated variables in a Principal Components Analysis (PCA). The Hypothesis column indicates whether an increase in a given variable is expected to result in an increase (+) or a decrease (-) in genetic connectivity. TPI = Topographic Position Index; TRI = Topographic Ruggedness Index; HF = Human footprint; Dstrb = Disturbance Category; Topo = Topography Category; For = Forest Category.

Variable	Category	Source	Climate PCA	HF PCA	Hypo-thesis	Description
Snow Days	Climate	NDVI^1^	●		**+**	Number of days with snow cover from NDVI NoData values (Fig. S5)
SWE	Climate	CHELSA^2^	●		**+**	30-year average (1981–2010) of snow-water equivalent with permanent water bodies removed
Temp	Climate	CHELSA^2^	●		**-**	30-year average (1981–2010) of annual maximum temperature
Building density	Dstrb	Micro-soft^3^		●	**-**	From all buildings in North America with point density tool in ARCGIS
Human footprint 1	Dstrb	NASA		●	**-**	8 anthropogenic variables: built-up environment, population density, electric power infrastructure, crop lands, pasture lands, roads, railways, navigable waterways
		SEDAC^4^				
Human footprint 2	Dstrb	Earth Systems Science Data^5^		●	**-**	14 anthropogenic variables: urban, crop land, grazing, mining, energy production (oil and gas, renewable), roads, railways, power lines, electrical infrastructure, logging, human intrusion, reservoirs, air pollution
HWY All	Dstrb	Street		●	**-**	Major Canadian and US Highways
		Maps^6^				
HWY 1	Dstrb	Street			**-**	North or south of Trans-Canada Highway 1
		Maps^6^				
Lights	Dstrb	NASA^7^		●	**-**	Nighttime lights
DEM	Topo	USGS^8^			**+**	30-m digital elevation model
TPI	Topo	USGS^8^			**+**	Topographic position index: From DEM using R terrain() in ‘raster’
TRI	Topo	USGS^8^			**+**	Terrain ruggedness index: From DEM using R terrain() in ‘raster’
Forest cover	For	NALCMS^9^			**+**	Proportion of cells in window of conifer or mixed forest type, 30-m
Forest edge	For	NALCMS^9^			**+**	Proportion of 30-m cells in window where conifer/mixed forest was adjacent to non-forested habitat

^1^https://modis.gsfc.nasa.gov. ^2^https://chelsa-climate.org. ^3^https://www.github.com/Microsoft/USBuildingFootprints. ^4^https://sedac.ciesin.columbia.edu/data/set/wildareas-v3-2009-human-footprint. ^5^https://zenodo.org/record/3963013#.YAG7DuhKiUk. ^6^https://openstreetmap.org. ^7^https://www.earthdata.nasa.gov/learn/backgrounders/nighttime-lights. ^8^https://www.usgs.gov/the-national-map-data-delivery/gis-data-download. ^9^http://www.cec.org/north-american-land-change-monitoring-system/.

## Results

**Fig. 2:**
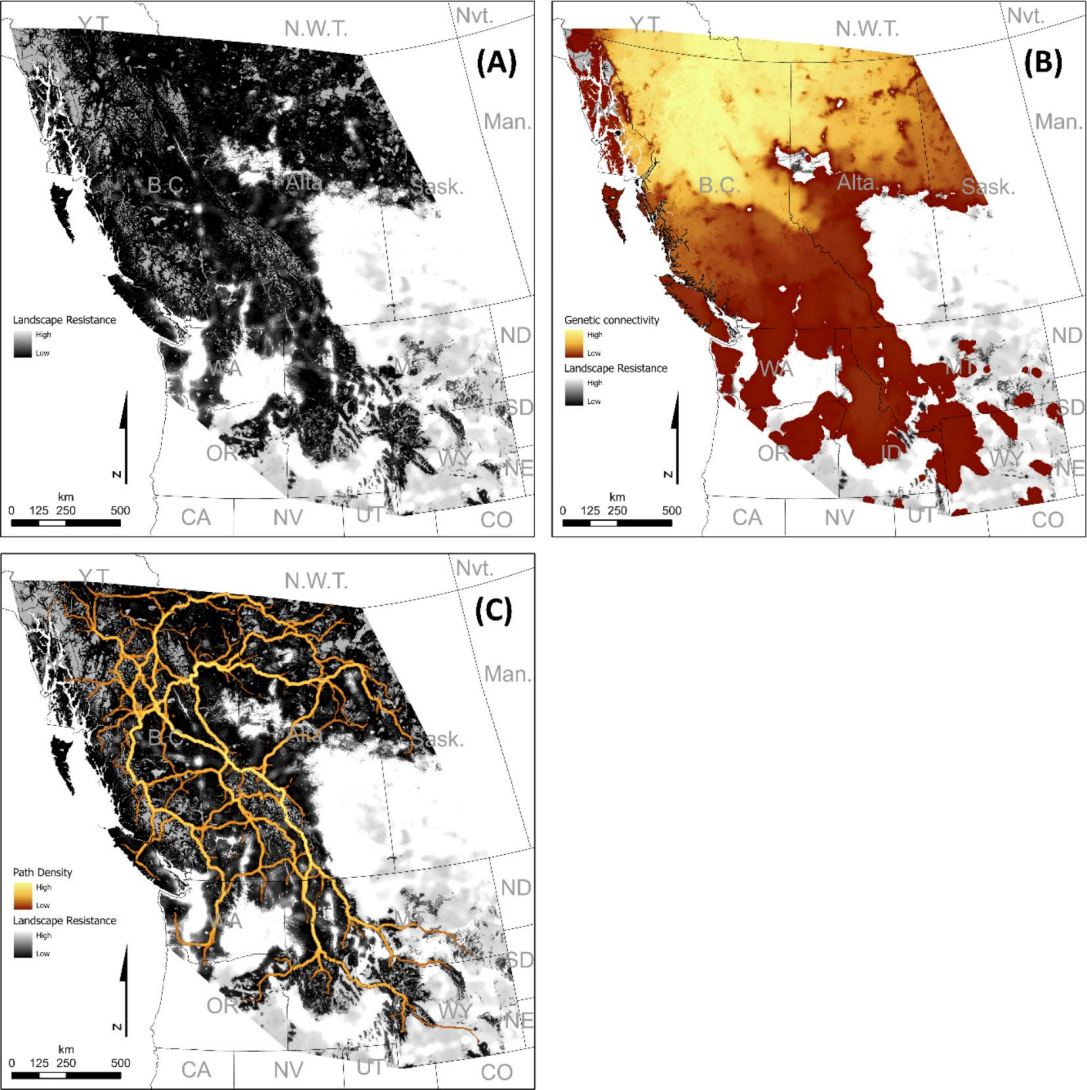
Genetic connectivity. Prediction surface for top performing model showing low (black) to high (white) landscape resistance due to human disturbance and lack of forest cover (**A**) with a resistant kernel (**B**) and factorial least-cost paths (**C**) indicating low (dark red) to high (yellow) genetic connectivity areas (**B**) and least-cost path density (**C**). These surfaces are inclusive of areas outside of current wolverine distribution to provide an understanding of how much resistance these areas pose to potential wolverine dispersal and connectivity.

Multiple statistical approaches converged on the conclusion that wolverine genetic connectivity across western North America was positively associated with forest cover and negatively associated with human disturbance. Genetic connectivity was greater in the northern than the southern extent of wolverine range, as was expected based on available habitat, past genetic studies, recolonization history, and inclusion of the periphery of the species’ range^28–30^. Overall, our results suggest that large areas of forested, snowy habitat with low human disturbance have likely facilitated recent gene flow via wolverine dispersal across western North America (Fig. 2, Supplementary Data 3). Multiple modeling approaches and validation techniques were used to corroborate these results as follows, with full details found in Supplementary Information.

### Spatial genetics

The relationships between genetic distance, geographic distance, and the logarithmic transformation of geographic distance are *r* = 0.290 and *r* = 0.321, respectively (Supplementary Fig. 1). A positive autocorrelation was observed up to 461 km with negative autocorrelation existing beyond 672 km (Supplementary Fig. 2). An interpolated value of 555 km as the threshold between positive and negative autocorrelation was chosen for subsequent analyses (i.e., *Scale-dependent results*). The spatial genetic diversity indices for estimates of allelic richness, inbreeding coefficients, and the effective number of breeding individuals within the 555 km spatial genetic neighborhoods are presented in Supplementary Fig. 3. Sampling density was disproportionately higher in the southern half of the study area, from southern British Columbia through northern Idaho and Montana (Supplementary Fig. 3A). We observed lower genetic diversity (allelic richness corrected by rarefaction; Supplementary Fig. 3B) and more inbreeding in the south than the north (Supplementary Fig. 3C). The northern-central portion of the study area had the largest relative effective number of breeders, decreasing towards the south and west (Supplementary Fig. 3D). In southeastern British Columbia, researchers found that the wolverine population was divided into several small areas of higher density bisected by corridors or areas with low to zero density^31^. In Idaho and Montana, USA, the estimated effective populations size was previously reported to be 28–52 individuals^13^, which aligns with our estimates of 19–51 (Supplementary Fig. 3D purple).

## Model optimization and relative importance of landscape predictors

In single-variable models of wolverine landscape genetic connectivity using maximum likelihood population effects analysis, the variables of human disturbance, forest cover, and snow days each outperformed the null model of geographic distance (Table 2). The best-performing multi-variable model included the human disturbance PC (upper exponential transformation, 1000 km^2^ window) and forest cover (lower exponential transformation, 1 km^2^ window; Table 2). Study-wide connectivity maps are presented in Fig. 2 and show areas of predicted low to high genetic connectivity for wolverines in western North America.

**Table 2:** Model selection results for maximum likelihood population effects model regression and least-cost transect analysis of wolverine genetic distances and landscape resistance based on least-cost paths. For maximum likelihood population effects models, the univariate step evaluated only univariate models and selected variables for the multi-variable step based on whether they outperformed geographic distance alone. For each variable, four moving window sizes and three transformations were tested, and the best performing combination is presented. Variables for the multi-variable step were produced by taking a weighted average across the variable rasters and recalculating least-cost paths. Distance and log of distance represent the null model of isolation-by-distance. AIC is used for ranking the maximum likelihood population effects models across both univariate and multi-variable models, and not for evaluating relative performance among models. For least-cost transect analysis, a gradient boosting machine was used to select features important to describing genetic distance based on average values along least-cost paths. TPI = Topographic Position Index; TRI = Topographic Ruggedness Index; PC1 = principal component analysis (axis 1); LCTA = least-cost transect analysis; MLPE = maximum likelihood population effects.

Variables	Window (km^2^)	Transformation	marg. *R*^2^	cond. *R*^2^	Δ AICc
**MLPE - Univariate**					
Human disturbance PC1	1000	upper exp	0.174	0.338	3002
Snow days	1000	linear	0.157	0.281	3438
Climate PC1	1000	lower exp	0.155	0.282	3774
Human footprint 1	1000	upper exp	0.197	0.375	3983
Log of distance	NA	NA	0.126	0.224	4814
Forest cover	1	linear	0.171	0.294	4854
Human footprint 2	1000	lower exp	0.139	0.247	6288
SWE	1000	linear	0.135	0.242	7205
Distance	NA	NA	0.139	0.245	8060
Building density	1000	lower exp	0.138	0.244	8074
TRI	1000	upper exp	0.141	0.248	8158
NASA lights	1000	lower exp	0.138	0.244	8164
Elevation	1	upper exp	0.140	0.245	8240
TPI	1000	upper exp	0.138	0.245	8243
Forest edge	1	upper exp	0.138	0.244	8261
All highways	NA	NA	0.091	0.211	17,660
Highway 1	NA	NA	0.057	0.158	28,962
**MLPE – Multi-variable**					
Human disturbance PC1 + forest cover			0.171	0.32	0
Human disturbance PC1 + snow days + forest cover			0.157	0.29	1105
Human disturbance PC1 + snow days			0.169	0.31	1533
Snow days + forest cover			0.167	0.29	3014
**LCTA – Straight line transect (Step 1)**					
Snow days + forest cover + geographic distance			0.194		
Human disturbance PC1 + snow days + forest cover + geographic distance			0.197		
**LCTA – Least-cost path line (Step 2)**					
Snow days + forest cover + geographic distance			0.1919		
Human disturbance PC1 + snow days + forest cover + geographic distance			0.1920		

## Consistency of top model

Because factors affecting landscape connectivity may vary at different spatial scales^32^, by sex and/or by region, we tested the robustness of results from the full data set to analyze subsets of data for each of these factors.

*Scale effects*. For finer-scale geographic distances ( < ~ 500 km), the same variables of forest cover (lower exp. transformation, 10 km^2^) and the human disturbance PC (lower exp. transformation, 1000 km^2^) were carried forward and outperformed all models when combined (Supplementary Table 1). For broader-scale geographic distances ( > ~ 500 km) the climate PCA produced the best-performing univariate model, but at the multi-variate step the best-performing model included forest edge (lower exp. transformation, 10 km^2^) and human disturbance PC (lower exp. transformation, 1000 km^2^).

*Sex Effects*. Male connectivity returned the same top model as in the full wolverine genetic dataset (Supplementary Table 2) for forest cover and human disturbance. However, geographic distance narrowly outperformed all models for females, suggesting that distance is more influential than the landscape variables tested. Individual splits by sex did not reveal statistically significant differences in spatial autocorrelation with females showing a positive spatial autocorrelation up to 435 km, negative spatial autocorrelation beyond 672 km, and Mantel correlation tests for isolation-by-distance (*r* = 0.288 [0.276,0.302]). Males displayed a positive spatial autocorrelation up to 467 km, negative spatial autocorrelation beyond 695 km, and *r =* 0.287 (0.274,0.299). For male genetic connectivity model optimization, the same three categories as in the global model were carried forward to the multi-variable step that included snow (linear transformation, 1000 km^2^), forest cover (linear transformation, 1 km^2^), and human footprint (lower exp. transformation, 1,000 km^3^). For female genetic connectivity, the logarithmic transformation of geographic distance outperformed all univariate and multi-variable models. We considered the second-best performing model of forest cover and human disturbance (Supplementary Table 2) to be competing with geographic distance (ΔAIC = 7 and higher R^2^ value).

*Northern and southern populations*. Finally, for both the northern and southern regional tests, the models with the variable of forest cover alone outperformed all other models (Supplementary Table 3). The northern population had lower genetic structure than the southern population (Mantel correlation tests with geographic distance; *r* = 0.216 [0.206, 0.227] versus *r* = 0.272 [0.258, 0.285], respectively). We repeated the maximum likelihood population effects model selection procedure on the two different sets of observations, thus assessing the influence of regional variation in habitat and landscape features on model inferences. Only forest cover (linear transformation, 10 km^2^) outperformed geographic distance in the less genetic structured northern population (Supplementary Table 3). For the more structured southern population, variables from all four categories were carried forward to the second step, including forest cover (lower exp. transformation, 1 km^2^), topographic ruggedness index (TRI; lower exp. transformation, 100 km^2^), human disturbance (linear transformation, 1,000 km^2^), and snow days (lower exp. transformation, 10 km^2^). However, forest cover alone outperformed all multi-variable models (Supplementary Table 3).

## Validating top genetic connectivity models

All 10 folds of the cross-validation procedure selected the same top model with forest cover and human disturbance, including scale and transformation, as in the full wolverine genetic dataset.

Repeating the maximum likelihood population effects model selection procedure for each independent landscape genetics simulation and comparing with the global ‘True’ model of forest cover and human disturbance PC resulted in a confusion matrix with an overall accuracy of 76.7%, sensitivity of 66.7%, and specificity of 81.0%. In summary, 6 of the 10 replicates selected the same top model as the global empirical model (human disturbance PC – upper exp. transformation, 1000 km^2^ and forest cover – lower exp. transformation, 1 km^2^), 3 of the 10 replicates selected global human footprint PC with snow days, and 1 of the 10 replicates selected the model with all three variables. No univariate model outperformed the multi-variable models for any of the 10 replicates.

## Assessing rigor of top genetic connectivity model with inter-model comparisons

The primary difference between the least-cost transect analysis and maximum likelihood population effects results was the retention of snow days in the majority of least-cost transect analysis’s top performing feature selection models. The top least-cost transect analysis model included snow days, forest cover, and human disturbance in addition to geographic distance and reported a root mean squared error = 0.077, *R*^*2*^ = 0.197, and mean absolute error = 0.060 (Table 2). The least-cost transect analysis feature selection modeling on 21 iterations of the spatial-fold cross-validations are reported for each of the 5 feature selection models in with the gradient boosting machine algorithm reporting slightly lower RMSE than (in ascending order) our generalized linear model, random forest model, generalized additive model, and linear model (Supplementary Fig. 4). The random forest model selected all variables 100% of the time during the training process and variable selection proportions for the other 4 models are shown in Supplementary Fig. 5. For the top performing algorithm (gradient boosting machine), two models were compared. The gradient boosting machine model with 3 variables (geographic distance, snow days, forest cover) reported a RMSE = 0.077, *R*^*2*^ = 0.194, and MAE = 0.061. The gradient boosting machine model with 4 variables (geographic distance, snow days, forest cover and human disturbance PC) reported a RMSE = 0.077, *R*^*2*^ = 0.197, and MAE = 0.060.

## Discussion

Genetic connectivity of wolverines in our study area was positively associated with forest cover and negatively associated with human disturbance with snow days receiving mixed support depending on the analysis. These environmental factors eclipsed geographic distance, despite the vast area across the extent of North American wolverine populations. Other regional-scale studies on wolverine landscape genetics have offered concordant conclusions^13,18^, although our continental-scale research placed stronger emphasis on forest cover and provided insights for a much larger spatial extent, addressed multiple regions, and used newer modeling techniques. This analysis represents one of the largest assembled collaborative datasets in terms of both geographic extent and number of wolverine genetic samples in North America. Below, we discuss these findings in the context of each landscape variable, landscape genetics methodology, and implications for conservation planning.

## Human disturbance

In our landscape genetics analysis, human disturbance was the strongest indicator of resistance to genetic connectivity (i.e., high resistance) for wolverines for all subsets of pairwise geographic distances using both modeling frameworks (maximum likelihood population effects models and least-cost transect analysis). This finding was also supported by genetic and habitat selection models at regional scales^12^. This result may suggest that protected areas with minimal recreation and development are important for promoting connectivity^22^. Of our variables tested describing human disturbance, those that consistently predicted genetic dissimilarity included composite variables with multiple factors such as road density, energy development, urban areas, and tourism. Non-composite variables describing more specific phenomena performed poorly, such as building density, nighttime lights (urbanization), and highways (movement barriers). Major highways correlated with population genetic structure in female wolverines at small spatial extents^33^ and induced avoidance behavior in terms of movement and habitat selection at the within-home range scale^22,34,,35^. It remains unclear how much of this result was due to effects of human disturbance on wolverine movements and the ecological changes that accompany development, including changes to prey, competitor, and predator communities. For example, humans tend to develop less rugged, less snowy areas in some regions, likely forcing a difference in habitat selection between humans and wolverine dispersers. However, while some cold and snowy areas of wolverine habitat are unoccupied by humans (e.g., high Rocky Mountains), others overlap with human recreation activities, are currently (e.g., arctic tundra), or were historically (e.g., Great Lakes region) occupied by both humans and wolverines. Furthermore, the landscape genetics analyses identified human disturbance as important when using the full data set, but not for either the north orsouth regions (Supplementary Table 3), suggesting that human disturbance may be more important to connectivity across larger distances. Overall, because of the differences from north to south, our results highlight benefits of conducting both focused regional and collaborative range-wide studies of wolverine’s genetic status and connectivity.

## Forest configuration (cover and edge habitat)

Previous habitat and genetic studies have suggested that (non-Arctic) wolverines select areas with high forest cover^36–39^ or prefer forest edge habitat^4,18,22,35,40^. We found that increased forest cover was associated with greater wolverine genetic connectivity. The positive response of forest cover was evident in regional analyses, as models with forest cover alone outperformed all other models for both north and south regions (Supplementary Table 3). Similarly, forest cover was positively associated with male and female genetic distances and least-cost paths (Supplementary Table 2). Forest cover was also an important variable for predicting genetic connectivity through least-cost transect analysis. At the finer scale for the maximum likelihood population effects analysis ( < ~ 500 km), genetic connectivity was partially explained by forest cover, while genetic connectivity at the broader scale distances ( > ~ 500 km) resulted in the only set of observations explained at least partially by forest edge, which included edge due to factors such as alpine timberline and forest fragmentation. In some regions dispersing wolverines may be attracted to forest openings and edge, presumably for prey resources and navigation, especially during particular seasons^35^. At the scale of our research, our analyses suggest that coniferous and mixed forest positively facilitated connectivity among wolverine populations. We interpret these results as evidence that forest cover may have increased opportunities for dispersing individuals and movement that facilitates genetic connectivity.

### Snow persistence

The extant wolverine fundamental niche has been generally characterized by cold, snowy areas^14,16,42^, though recent studies from North America^41^ and Scandinavia^25^ have questioned the importance of persistent snow cover for wolverine denning. Evidence that both snow persistence and snow depth promote wolverine genetic connectivity has been reported previously^13,18^ in studies that were limited to the southeastern periphery of the wolverine’s North American range where snow is more limited and ephemeral than at higher latitudes. Studies of habitat use within home ranges have reported similar patterns of selection for snow persistence^21,24,27^; however, habitat suitability models have often been unsuccessful at predicting genetic connectivity^44,45^. Our results provide some support for the hypothesis that snow persistence was important for wolverine genetic connectivity, even if this variable was not in our top maximum likelihood population effects model. Snow or snow-water equivalent was retained in all of the least-cost transect analyses modeling algorithms, and snow was retained more than forest cover when using the top-performing least-cost transect analysis algorithm (Supplementary Figs. 4, 5). This relationship was captured by the nonlinear machine learning models within the least-cost transect analysis algorithm but not the linear regression model of the maximum likelihood population effects method and may highlight a difference in capability between the two methods^43^. Snow days outperformed geographic distance in most maximum likelihood population effects univariate analyses (Supplementary Tables 1–3), emerged as the second-best model from the full dataset, and the final genetic connectivity prediction surfaces were highly correlated with the top model (Supplementary Fig. 6, Table 1). In addition, model results from the simulated wolverine populations (see Methods - *Landscape resistance model validation tests*) selected snow days in place of forest cover in 3/10 model runs, and snow days along with forest cover in 1/10 model runs, suggesting that fewer snow days was likely collinear with lack of forest cover, though resistance associated with fewer snow days was correlated with resistance due to human disturbance as well (Supplementary Table 4). Overall, our results showed that climate factors were not the only suite of variables influencing wolverine connectivity.

### Scale and shape of environmental variables

Wildlife behavior and genetic processes respond to environmental features occurring at variable spatial scales^32,46^. Accounting for this scale-dependent variation increases our ability to accurately describe how animals select habitat at multiple scales (e.g., within and among home ranges, during dispersal), and how these decisions translate to gene flow across large distances^47^. Ultimately, genetic connectivity is the result of wolverines making fine-scale dispersal movement decisions that accumulate to produce larger dispersal distances, and over time these behaviors accumulate to produce large-scale population connectivity. Our multi-scale investigations revealed that wolverine connectivity was influenced simultaneously by both fine-scale vegetation factors and broad-scale influences of human impact and climatic factors. We performed four different analyses to understand how issues of the scale and shape of environmental variables affected our results. First, environmental resistance variables included in the maximum likelihood population effects analysis were sampled at four scales to identify the optimal scale at which to capture the resistance of the variable to gene flow. With this first approach, we found that most environmental resistance variables influenced genetic connectivity to a greater degree at the largest scale we measured (1,000 km^2^), although forest cover and terrain variables tended to perform better with the finer spatial scale (≤ 10 km^2^). Second, we tested multiple transformations (i.e., shape) to allow for nonlinear relationships between each variable and its resistance to gene flow, which improved model fit and accuracy. Third, because of the differences in genetic diversity and habitat characteristics from the northern to the southern extent of the study area, we conducted regional analyses on this north-south gradient to test for the spatial robustness of model results and account for non-stationary factors^48^. Finally, we conducted analyses on different subsets of geographic distance among wolverines, which also resulted in identification of different variables associated with genetic similarity. Despite the well-known effect of scale on habitat use, adoption of multi-scale analyses of landscape genetic connectivity has lagged^18,47,49^. In the accumulation of our analyses, our results demonstrate the benefit of evaluating multiple scales, particularly for landscape connectivity studies on wide-ranging species. For conservation and management, we interpret our results as demonstrating practical applications where we acknowledge the effects of scale on the variables that best explain genetic connectivity.

### Landscape genetics methodology, validation, and importance of inter-model comparison

Our model validation and evaluation efforts^50^ served to strengthen the reliability of our methods and results, but have also produced insights about the potential relationships among variables such as forest cover and snow days. We used two different approaches as a means of inter-model comparison to test for model robustness and performance and improve confidence in our conclusions^51^. We have shown that when combined with machine learning approaches^43,49,52^, the least-cost transect analysis produced similar results to the pseudo-optimization routine for the maximum likelihood population effects modeling and provided additional information. For example, the best performing least-cost transect models retained snow days as positively associated with wolverine genetic connectivity, whereas the multi-variable maximum likelihood population effects models did not. It is possible that least-cost transect methods combined with machine learning are better equipped to account for interactive and non-linear effects^43^. As statistical models for landscape genetics continue to be refined and developed, it is critical that multiple methods are used to validate results and to glean insights based on differences among model outcomes. We also employed other means of validating and evaluating our results that strengthened the evidence in favor of our statistical approaches, such as cross-validation and simulation modeling. These measures, such as cross-validation and simulation modeling to test model performance, are needed in fields where methodology is rapidly developing and debate continues about the robustness of those methods to the diversity of applications in which they are applied^53,54^. Differences in results that emerge across models spur alternative hypotheses and guide future research that may elucidate reasons for the discrepancies. In the present case, for example, we expect there could be different interpretations with higher genetic resolutions, such as what could be gained from single nucleotide polymorphism panels instead of microsatellite markers^55^.

### Implications for transboundary conservation and management of wolverines

In southwestern Canada and the northwestern contiguous United States, wolverines have large home-ranges (500–1,000 km^2^) that cross national, tribal, provincial, and state boundaries; consequently, effective conservation efforts will require cooperation among many levels of government. We provided the first landscape genetics analysis spanning the transition zone from the Canadian and Alaskan populations in the north to the southern populations in the coterminous United States. Our study enables consideration of suitable management actions at large spatial scales and can serve as a baseline against which to compare future genetic patterns as landscapes change. Our results indicated high levels of landscape genetic connectivity across the wolverine’s range in much of western North America, with a clear signal of high-to-low genetic diversity and connectivity from north-to-south, corroborating past literature^28,56^. The covariates that parsimoniously explained wolverine genetic connectivity reflect that gene flow occurred across large areas of southwestern Canada, with populations becoming more fragmented towards the southern extent of the range. This pattern may be partially due to the fact that wolverines were extirpated from and have recolonized multiple southern portions of the study area since the mid-20th century^30^.

Species conservation for wolverines hinges largely on large-scale landscape management with connectivity increasing with forest cover, less human disturbance, and persistent snow cover^57^. Based on our results, minimizing anthropogenic landscape development or forest removal in regions between areas with predicted wolverine occurrence would promote gene flow. Understanding how our predicted least-cost corridors intersect with existing and planned land-use change and transportation infrastructure is the first step to provide science-based management actions and conservation planning within the wolverine’s range in North America. Additional work will be needed to understand how localized projects that affect forest cover and human disturbance could impact potential dispersal pathways of wolverines, particularly in areas where genetic connectivity is more fragmented. Regardless, the large spatial extent of wolverine habitat combined with long-distance dispersal behavior requires that cumulative effects of such projects need to be considered at multiple spatial scales. Future analyses should focus on utilizing the results of this genetic connectivity study to identify existing and potential key habitat linkages between wolverine core habitats. Our study builds on a limited but growing foundation regarding wolverine genetics. We achieved relevant and timely assessments from the collaboration of researchers, landowners, and managers within two countries, resulting in the first wholistic description of genetic relatedness and connectivity for North America’s wolverines.

### Methods

#### Study system

The 2,173,501 km^2^ study area encompassed most of the western Canadian provinces of British Columbia and Alberta, southeastern Alaska, and the currently occupied wolverine habitat in the contiguous United States in Montana, Idaho, Wyoming, and Washington (Fig. 1). This transboundary study area contains diverse wolverine habitat from montane ecosystems in the south to boreal forests in the north.

### DNA sample collection and analysis

We collected 882 multi-locus genotypes (489 males, 393 females) using 19 loci for the landscape genetic analysis (data available upon request). These wolverine genetic data were extracted from hair snagging, live-capture, and trapper harvest from a group of > 40 wildlife researchers and managers between 2006 and 2016 across Alberta, British Columbia, Idaho, Montana, Washington, Wyoming, and Southeast Alaska (Fig. 1). All DNA extractions (using QIAGen^58^ DNA extraction kits) and genetic analyses were performed at Wildlife Genetics International (WGI) in Nelson, BC, and the National Genomics Center for Wildlife and Fish Conservation at the Rocky Mountain Research Station (RMRS) in Missoula, MT. The aggregated dataset contained 882 complete and unique genotypes (489 males, 393 females) for the landscape genetic analysis. Summary statistics for the 19-microsatellites from GenAlex^59^ included mean number of alleles = 6.3 with SE = 0.42, total alleles, *Na* = 118, and Fixation Index = 1 - observed heterozygosity / expected heterozygosity = 0.078 with SE = 0.008 (Supplementary Table 5), as well as tests for neutrality (Supplementary Fig. 9).

### Individual-based genetic differentiation

To estimate genetic distance among individuals, we used the software Spagedi^60^ and the R package ‘gstudio’^61^ to calculate several metrics that were recommended in a review of genetic distance measures for landscape genetics^62^. These metrics included kinship coefficients^63,64^, relationship coefficients^65–68^, Rousset’s A^69^, proportion of shared alleles^70^, and Euclidean distance^71^. All metrics were highly correlated, *r* > 0.92, which can result in non-robust resistance surface optimization^72^. To simplify the various metrics into a single distance measure, we conducted principal components analysis across the pairwise matrices of genetic distance and used the first principal component as our measure of genetic distance for all landscape genetics analyses. We provide genetic distances for all subsets in Supplementary Data 1.

### Spatial genetic structure

Mantel tests for Pearson’s correlation and spatial correlograms (‘ecodist’ package in R^73^) were used to assess the extent of spatial genetic structure and scale effects in the wolverine data set^74^. We used the ‘sGD’ package in R^75^ to estimate genetic diversity based on grouping individuals into overlapping genetic neighborhoods. We inferred a genetic neighborhood based on the correlograms that depict the spatial autocorrelation of genotypes^76^. This approach has the advantages of being able to capture spatially complex patterns of genetic diversity in clinal or landscape-driven populations while not having to dictate population boundaries a priori. Within each neighborhood, we calculated estimates corrected for sample size of allelic diversity^77^, inbreeding coefficient^78^ (or Fixation Index), and effective number of breeders^79^ (Wright’s neighborhood size) using NeEstimator^80^ within ‘sGD’, as well as sample size within the genetic neighborhood^81^.

### Landscape variables and curation

We identified hypotheses for how landscape variables likely impede or promote genetic connectivity for wolverines in North America (Table 1). Selection of landscape variables was based on existing literature and an expert opinion working group of collaborators. We assigned the selected variables to one of four categories for how genetic connectivity will be affected, that is, hypothesized to be negatively affected by human disturbance, climate, topography, and vegetation. We curated a set of spatial datasets to represent each variable in terms of resistance to wolverine movement by using three transformations and four spatial scales of each resistance surface^18^ (described below; Table 1) to represent alternative sensitivity to landscape resistance and spatial scale. All variables can be obtained publicly (but see snow days Table 1 and Supplementary Fig. 7). To ensure consistency of variables across the transboundary study extent, raster datasets were only obtained from sources that covered both the United States and Canada. To minimize distortion of spatial data projected across such a large study extent, we reprojected all raster data to a custom azimuthal equidistant projection centered within the study area.

### Landscape resistance hypotheses

Landscape resistance surfaces are characterized by geospatial rasterized cells in a digital layer where each cell is given a relative value reflecting the impediments or barriers to gene flow. We rescaled all variables to standardize environmental predictors (Table 1)^18^. First, we resampled all raster grids to 1 km^2^ resolution because this resolution was broad enough to make the data analysis computationally feasible, and is an appropriate minimum scale given the daily movements of wolverines, this resolution was unlikely to influence biological or computational outcomes. Then, we rescaled raster values between 0 and 1, representing the minimum and maximum resistance present within the study area (raster cells with highways were assigned a value of 1, with all other values 0). We used three transformations on these rescaled values^43^ to represent variable sensitivity to resistance following a weighted distance approach^82^: linear, upper exponential, and lower exponential (e.g., upper exponential transformations represent high sensitivity to changes at low values of resistance and vice versa^18^). Finally, because the scale at which animals respond to stimuli and the scale of dispersal habitat needed to survive a dispersal event can vary across variables, we used a moving window analysis to capture each variable at 4 different scales with windows of 1-, 10-, 100-, and 1,000 km^2^. Once the complete set of resistance surfaces was created, we used the costDistance() function in the ‘gdistance’ package in R^83^ to calculate effective distances between all pairs of individual wolverine observations. The resulting resistance or effective distance matrices were used as explanatory variables in the landscape genetics analyses (lower triangle of the full dataset; 882 × 882 matrix or 882 * 881 / 2 = 388,521 pairwise observations; Supplementary Data 2). No transformations or moving windows were run on the highway rasters, as these represented linear barriers.

### Linear mixed models for spatial genetics

Statistical modeling techniques have emerged over the last few decades to test for individual (or population) measures of genetic connectivity relationships, and many criticisms have followed from subjectivity of resistance surfaces to methodology issues associated with spatial autocorrelation^84,85^. We used maximum-likelihood population-effects, a mixed modeling regression approach that treats the residual for each pairwise distance as the sum of two random population-level effects and an observation-level error^86^. Because the maximum likelihood population effects correlation structure allows modelling the non-independence of pairwise distances within a likelihood framework, compatible with model selection, it is particularly appealing for landscape genetic studies^87^. The fixed effects are the pairwise effective distances from each landscape resistance hypothesis, and the random effect utilizes a covariance structure to account for the correlation between pairwise data points. Maximum likelihood population effects modeling has outperformed multiple regression with distance matrices and causal modeling with Mantel tests^53^ while accounting for autocorrelation structure inherent in all distance-based analyses^88^. Prior to analysis, we evaluated all pairs of variables for multicollinearity (Supplementary Table 4) and excluded one variable out of any pair with a Pearsons’ correlation value > 0.7^89^. Snow days and maximum annual temperature were the only two variables that exceeded this correlation value, and therefore we excluded maximum temperature. We used a two-step hierarchical model selection procedure to identify the best model for predicting genetic distances among pairs of wolverines (i.e., pseudo-optimization^89^). While optimization methods for maximum likelihood population effects models exist (e.g., ‘ResistanceGA’^90^), these are computationally demanding and were unable to accommodate the combination of landscape size and number of observations in our data set, as well as recent evidence pointing to unreliability of results^72^. In the first step of model selection, we ran univariate maximum likelihood population effects models for each combination of variable, transformation, and scale (e.g., snow days, linear transformation, 10 km^2^). The best scale-transformation combination for each category of variables (climate, human disturbance, topography, forest) that outperformed log-transformed geographic distance was carried forward to the second step. Distance-only models represent the null model of isolation-by-distance because they assume that landscape patterns have no effect on genetic connectivity. In the second step of model selection, we created a candidate set of multi-variable models by combining variables that were carried forward from the first step^92^. Rasters for each variable in a candidate model were combined into a single raster using weighted averaging, where the weight a variable received was equal to 1/n, with n equal to the rank of that variable from the univariate step. We averaged resistance across explanatory variables instead of using multiple regression on multiple distance matrices because least-cost paths associated with different environmental variables can take widely different routes across a landscape^92^. We tested all possible 2- and 3-variable combinations and included all 3 transformations for each variable to allow for the possibility of nonlinear associations among variables to improve performance under different transformations. We repeated the method used in step 1 to create effective distance matrices and conducted AIC model selection on the full set of univariate and multi-variable models. Model performance was judged primarily on Akaike’s information criterion (AIC), which is suitable for maximum likelihood population effects models that don’t use restricted maximum likelihood^62,88,91^.

#### Landscape resistance model sensitivity tests

*Scale effects*. Because factors affecting landscape connectivity may vary at different spatial scales^32^, we tested for scale effects in the full wolverine data set using the spatial autocorrelograms genetic neighborhood cutoff value^18^ (Supplementary Fig. 2; ~555 km). Based on these results, we partitioned the data set into two groups: one including only pairwise comparisons less than or equal to a geographic distance of 555 km (“fine scale”) and the other including only those pairs of individuals with geographic distances greater than 555 km (“broad scale”). We then repeated the maximum likelihood population effects procedure at the two geographic scales, thus assessing the influence of scale effects on model inferences.

*Sex effects*. We split the wolverine genetic distance data set by male and female. We then repeated the maximum likelihood population effects procedure for the two data sets to assess the effect of sex on model results.

*Northern and southern populations*. Wolverines in North America have greater genetic structure in the southern periphery of their range than in regions further north^29^. Two landscape genetics studies in the southeastern population have shown support for spring snow cover, snow depth, buildings, and terrain ruggedness^13,18^. Therefore, to identify regional differences in connectivity, as well as the ability to model features that may only serve as limiting factors in one region^48^ (e.g., snow days in the south), we conducted maximum likelihood population effects analyses on north and south data sets (“northern” *n* = 403 vs. “southern” *n* = 479 individuals). Observations were grouped as either north or south of Highway 1 in Canada, which we hypothesized was a potential barrier to female wolverine connectivity^33^.

### Landscape resistance model validation tests

*Random cross-validation of predicted genetic distance*. To test for consistency in maximum likelihood population effects model selection results, we conducted 10-fold cross-validation by randomly withholding 20% of the individuals and training the model on the remaining 80%. We used the same candidate set of models that was used on the full data set, and recorded the number of times that maximum likelihood population effects analyses selected the same model as the full data set. Using the selected model from each training data set, we predicted genetic distances for the remaining 20% of observations and recorded the root mean squared error (RMSE).

*Individual-based genetic simulations*. Landscape genetics models and methods have been subject to uncertaintines in their ability to have enough power to identify the correct processes driving gene flow^95,99^. To formally evalute our statistical approach and further corroborate results, we used simulations to stipulate the actual species-and individual-specific processes in action and to test the reliability with which MLPE was capable of identifying the correct resistance models i (pattern-process or pattern-oriented modeling^93^). We used the individual-based landscape genetics simulator (CDPOP v1.3.15^94^) to (1) test the ability of the resistance surface to reproduce empirical genetic patterns, and (2) test our ability to correctly attribute the causes of observed genetic structure with the statistical methods used here^95^. This approach provides a controlled simulated environment and helps support empirically derived results of observed genetic patterns. We therefore generated genetic data across individuals in a spatially distributed pseudo wolverine population over time as a function of the landscape resistance hypothesis produced from the empirical landscape genetics analyses that used the full data set described above. Using the empirical derived landscape resistance surface, we placed 1,000 spatially weighted points (i.e., low resistance cell values received a higher probability). We then calculated the pairwise effective distances between the 1,000 points with the landscape resistance surface using the costDistance() function in the ‘gdistance’ package in R^83^. These effective distance values were used to specify dispersal and mating probabilities based on an inverse-square distribution with 30% maximum movement of the total landscape’s effective distance (see Supplementary Fig. 8). This movement distribution was chosen through initial exploratory simulations varying both functional form and maximum movement, with the goal of calibrating the model to achieve realistic patterns of dispersal behavior and genetic diversity over time. Each simulation tracked 19 loci with an initial starting value of 30 alleles per locus randomly assigned to the first generation (the same number of total loci as our empirical data set) in a female without replacement and male with replacement mating structure for 300 non-overlapping generations. Each mate pair produced a number of offspring following a Poisson process such that population growth was kept constant. After ~ 25 generations, simulations with these parameters converged on a fixation index of 0.08 (the approximate population genetics structure of the empirical data set). We produced 10 replicates and within each replicate chose a generation time to analyze such that the maximum starting alleles of 570 decayed to 118 alleles (total alleles within empirical data set). The replicate generation times that matched both *F* = 0.08 and *Na* = 118 varied and occurred between 260 and 290 generations. Finally, each replicate with 1,000 individual genotypes was used to repeat the maximum likelihood population effects process described above and a confusion matrix (ConfusionMatrix() function in the ‘caret’ package in R^96^) was used to determine how many times the underlying landscape resistance surface was recovered (i.e., total accuracy, specificity, sensitivity).

#### Landscape resistance inter-model comparisons

*Model comparison with least-cost transect analysis*. Least-cost transect analysis or corridor-based approaches^91^ extract summarized values of covariates along straight-line or least-cost path lines that are then used to predict genetic distances. Optimization procedures^97^ with a maximum likelihood framework were introduced with more recent studies adding random forest models^52,98^. Applications of least-cost transect analyses were expanded to individual-based samples using gradient boosted regression models with spatial cross validation^49^. Machine learning approaches are beginning to show promising results for landscape genetics and may possibly improve predictive power^99^. However, inter-model comparisons ultimately provide improved reliability in prediction results, if many models converge to similar results.

Here, we applied the individual-based, machine learning, least-cost transect analysis methodology^49^. First, we extracted the mean value along a 3 km buffered straight line (results were not sensitive to the size of buffer) between all pairwise locations for all environmental variables using the exactextract() function in the ‘exactextractr’ package in R^100^. The covariates extracted from these straight lines along with geographic distance (to minimize spurious results^95^) were then used as explanatory variables with genetic distance as the response variable. Prior to modeling, we used k-means clustering to assign observations to distinct spatial groups for use as cross-validation folds. We created groups of both k = 10 and k = 20 clusters, to evaluate the effect of different spatial structures on model selection results. Spatial clusters were assigned such that groups of individuals and all connections to other individuals were withheld at each fold (approximately 22% of the data). We then applied a forward feature selection algorithm in the ‘CAST’ package in R^96^ using 5 different model types or algorithms: gradient boosting machine^101^, random forests^102^ (‘ranger’ package in R^103^), generalized linear elastic-neural network model^104^, generalized additive model, and a linear model. For each model type, we performed 21 feature selection replicates using the groups of k = 10 or a unique combination of k = 20 clusters. The optimal predictor set for each forward feature selection replicate was retained and then summarized across model types. GBM errors were lowest among the 5 model types and we continued using only GBM. Using the model trained using straight line variables, we created a prediction surface using the ‘raster’ package in R^105^. We then used this straight line generated genetic connectivity prediction surface to create least-cost path lines using the paths() function in the ‘gdistance’ package in R. Landscape variables were then extracted along each path and used as predictors in a second stage of model training to determine the best final least-cost transect models.

#### Study-wide connectivity

We used the software UNICOR^106^ to map predicted genetic connectivity for the final resistance genetic connectivity surface. We used two connectivity algorithms to highlight probable dispersal corridors: (1) factorial least-cost paths (Dijkstra’s algorithm^107^) and (2) resistant kernels^108^. Given an input resistance surface, factorial least-cost paths calculate all potential routes for gene flow between pairs of source points and highlight optimal dispersal paths, while resistant kernels depict corridors or movement areas that are smoothed as a function of cumulative cost and corridor intensity^109^. For both factorial least-cost paths and resistant kernels, we used 1,000 randomly spatially weighted source points (higher probability of spatial location in lower resistance values) within the study area. For factorial least-cost paths, we did not specify a dispersal threshold in effective-distance units. For resistant kernels, we used the volume calculations (‘Kernel_volume’ = 5,000 and ‘Const_kernal_vol’ = FALSE) with ‘Transform_function’ = linear across an ‘Edge_Distance’ = 5,000.

## Electronic Supplementary Material

 Below is the link to the electronic supplementary material.

Supplementary Material 1

Supplementary Data 1

Supplementary Data 2

Supplementary Data 3

## Data Availability

The datasets generated to replicate the landscape genetic analyses are available in the Supplementary Information as Supplementary Data 1-2. Raw genotypes with locations are available by request to author MAS at sawaya.mike@gmail.com with collaborator approval. Supplementary Data 3 provides files for visualization of the connectivity landscape.

## Code availability

All code to replicate these analyses provided upon request.
