# Supplementary material for: Genetic connectivity of wolverines in western North America
Source: Sci Rep. 2024 Nov 15;14:28248. doi: 10.1038/s41598-024-77956-9 (PMC11568290; doi:10.1038/s41598-024-77956-9)
Supplement: Supplementary file 1 — Supplementary Material 1 [file 41598_2024_77956_MOESM1_ESM.docx]

**Supplementary Information for**

Genetic connectivity of wolverines in western North America

**Authors**

Casey C. Day^1*†^, Erin L. Landguth^1*†^, Michael A. Sawaya^2^, Anthony P Clevenger^3^, Robert A. Long^4^, Zachary A. Holden^5^, Jocelyn R. Akins^6^, Robert B. Anderson^7^, Keith B. Aubry^8^, Mirjam Barrueto^9^, Nichole L. Bjornlie^10^, Jeffrey P. Copeland^11^, Jason T. Fisher^12^, Anne Forshner^13^, Justin A. Gude^14^, Doris Hausleitner^15^, Nichole A. Heim^16^, Kimberly S. Heinemeyer^17^, Anne Hubbs^18^, Robert M. Inman^19^, Scott Jackson^5^, Michael Jokinen^7^, Nathan P. Kluge^14^, Andrea Kortello^20^, Deborah L. Lacroix^21^, Luke Lamar^22^, Lisa I. Larson^13^, Jeffrey C. Lewis^23^, Dave Lockman^24^, Michael K. Lucid^10,11^, Paula MacKay^5^, Audrey J. Magoun^25^, Michelle L. McLellan^26^, Katie M. Moriarty^27^, Cory E. Mosby^11^, Garth Mowat^28^, Clifford G. Nietvelt^28^, David Paetkau^29^, Eric C. Palm^1^, Kylie J.S. Paul^30^, Kristine L. Pilgrim^5^, Catherine M. Raley^8^, Michael K. Schwartz^5^, Matthew A. Scrafford^31^, John R. Squires^5^, Zachary J. Walker^24^, John S. Waller^32^, Richard D. Weir^33^, Katherine A. Zeller^5^

**This PDF file includes:**

Supplementary Figures 1 to 9

Supplementary Tables 1 to 5

Supplementary Data 1 to 2

**Supplementary Figures 1 – 9**


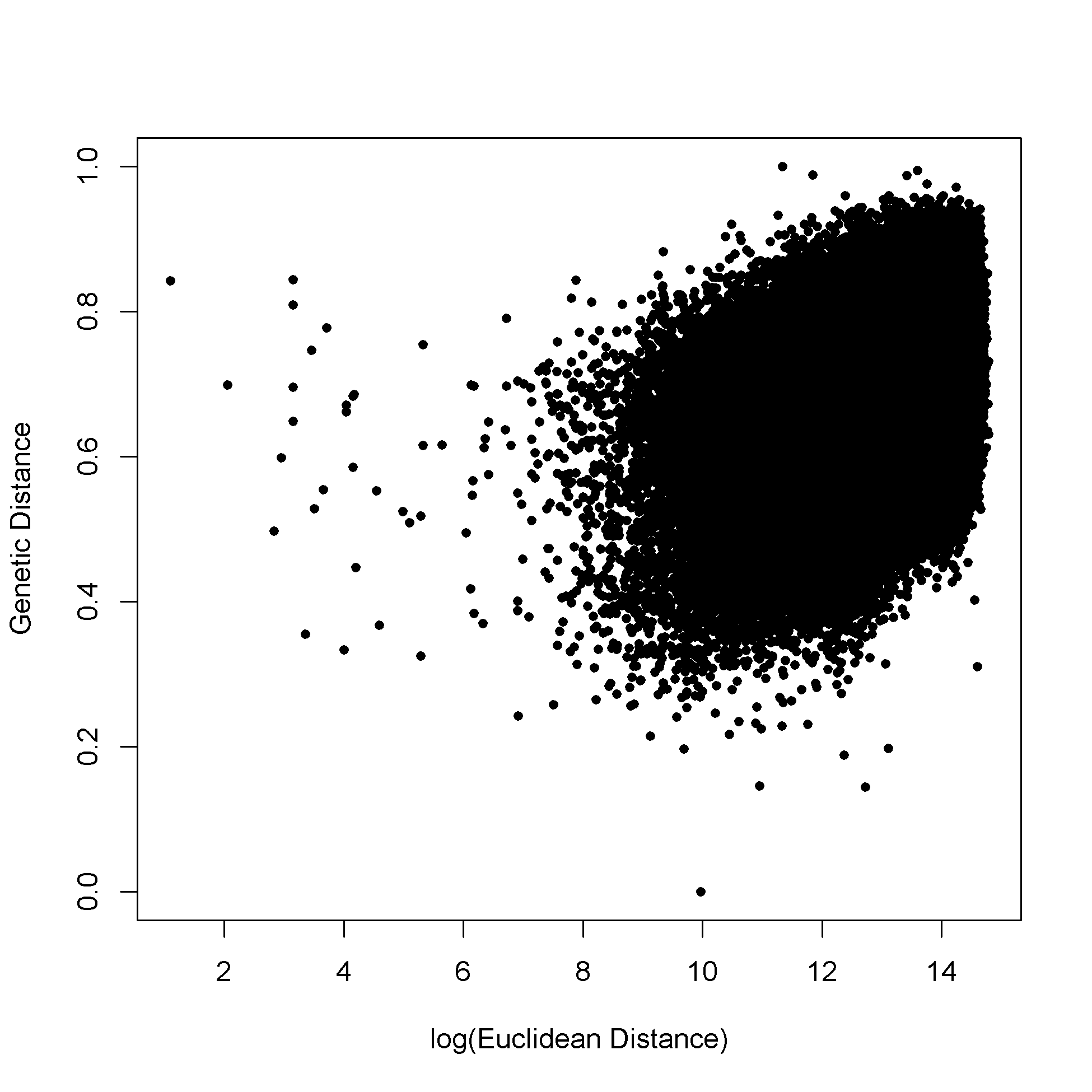

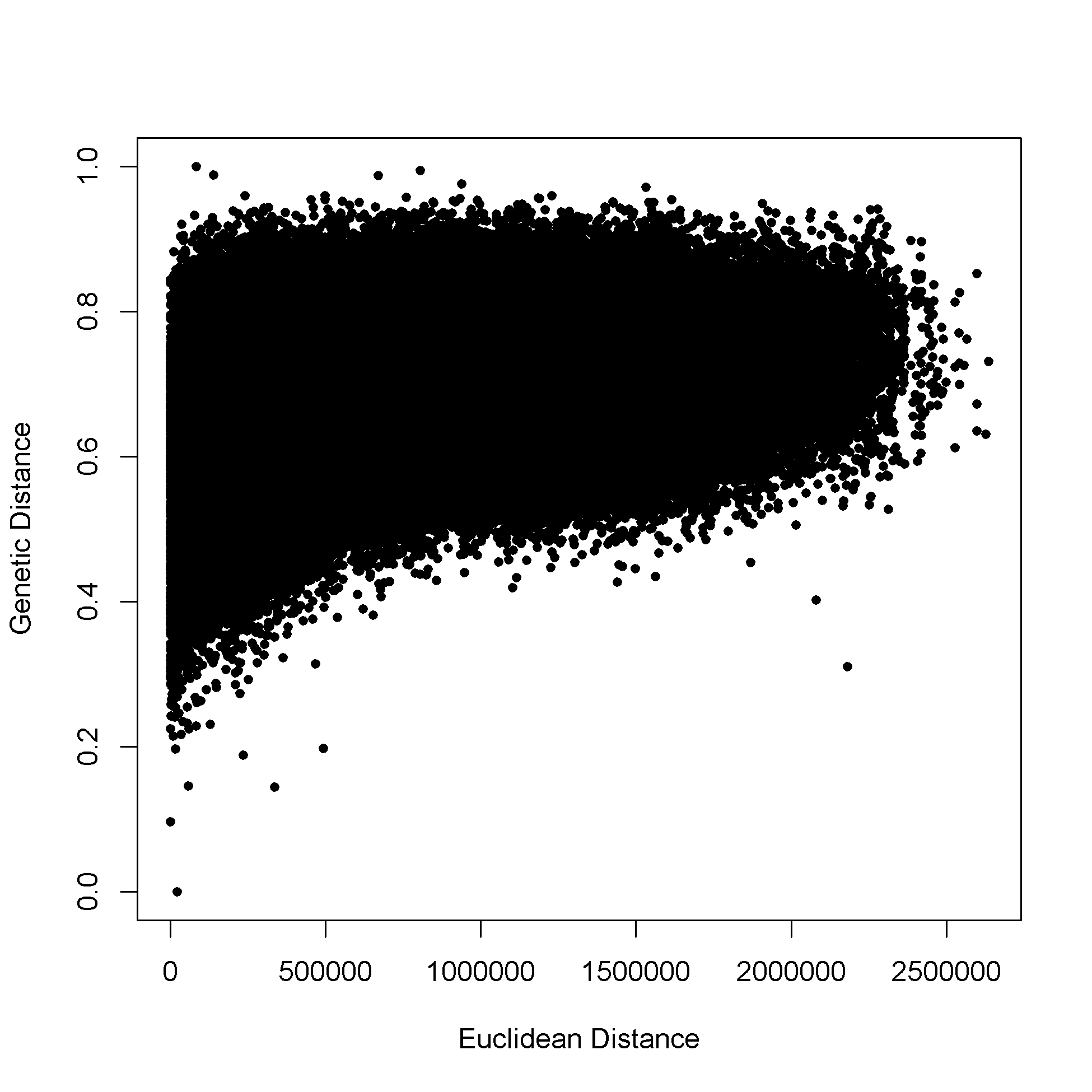


**(A)**

**(B)**

**Supplementary Figure 1: Genetic distance versus geographic distance.** Untransformed shown in (A) with *r =* 0.290 and logarithmic transformed shown in (B) with *r* = 0.329 (P-values < 0.001). The genetic distances used here are the values from the first axis of a principal components analysis run across several individual-based genetic distance metrics that were standardized – and are therefore unitless. Euclidean distance is in meters.

**
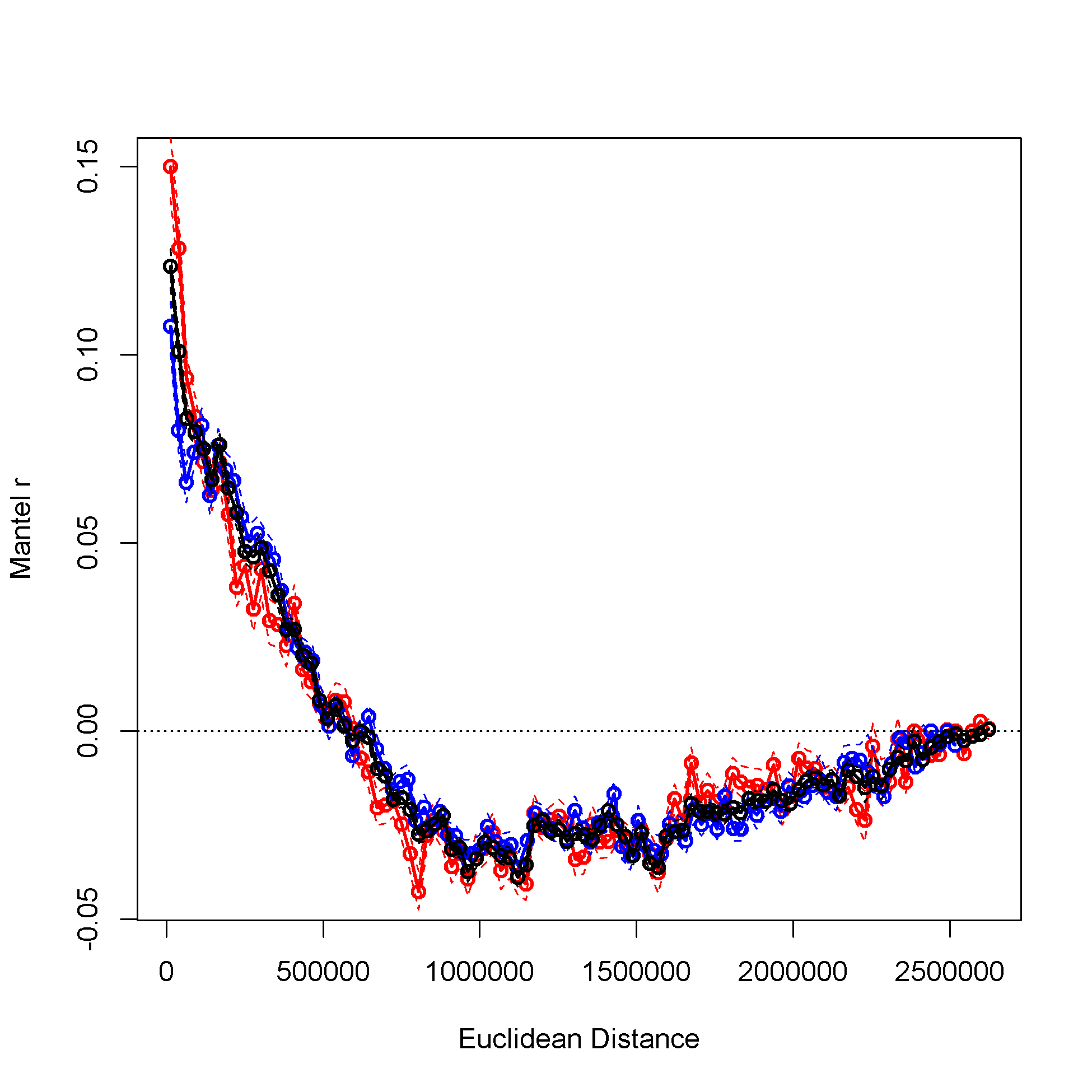
**

**Supplementary Figure 2: Spatial autocorrelation.** The spatial-genetic extent of positive autocorrelation for all individuals was < 555 km. Y-axis is geographic distance. All samples (black), male samples (blue) and female samples (red).


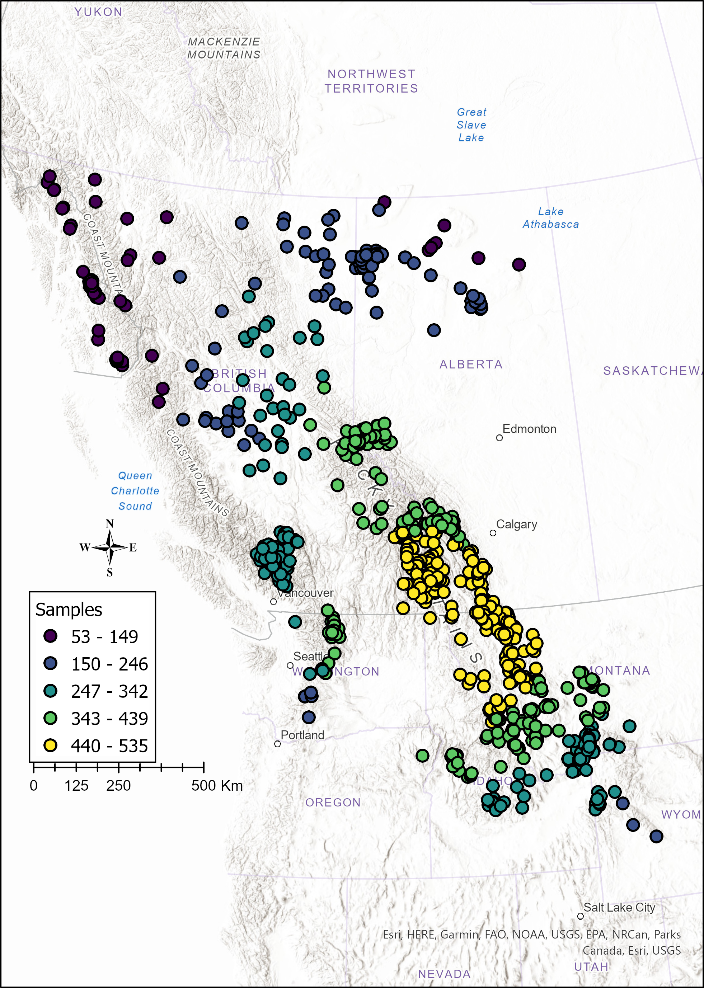

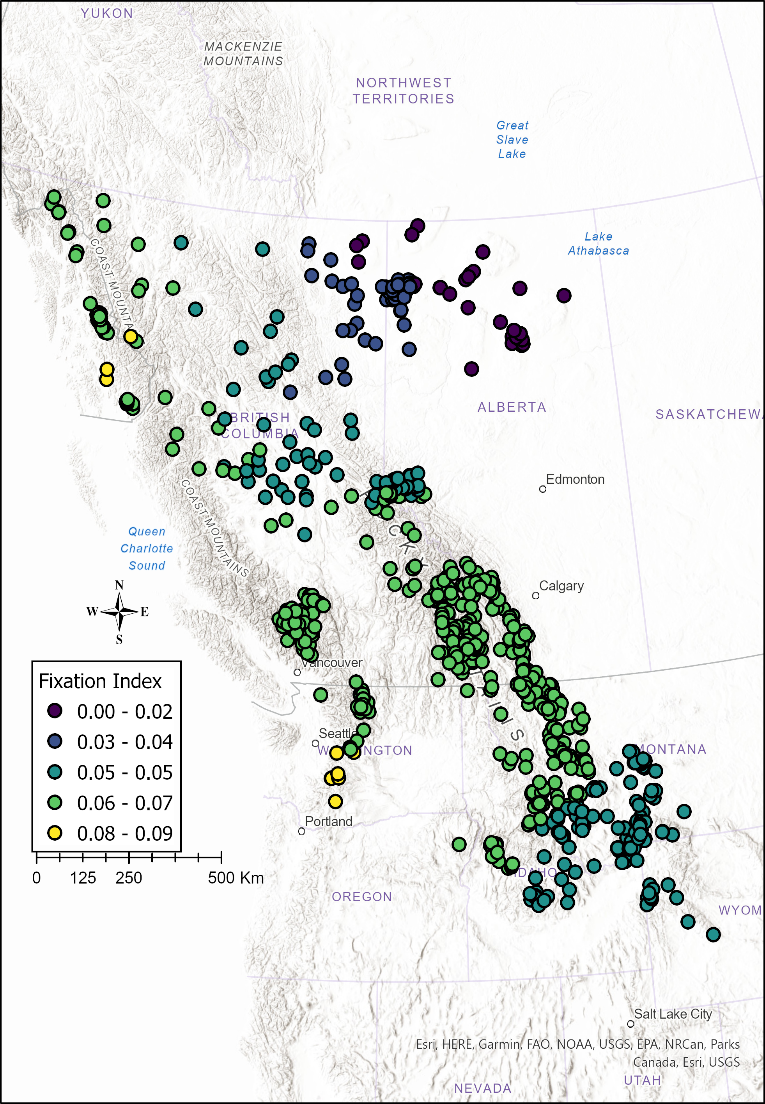

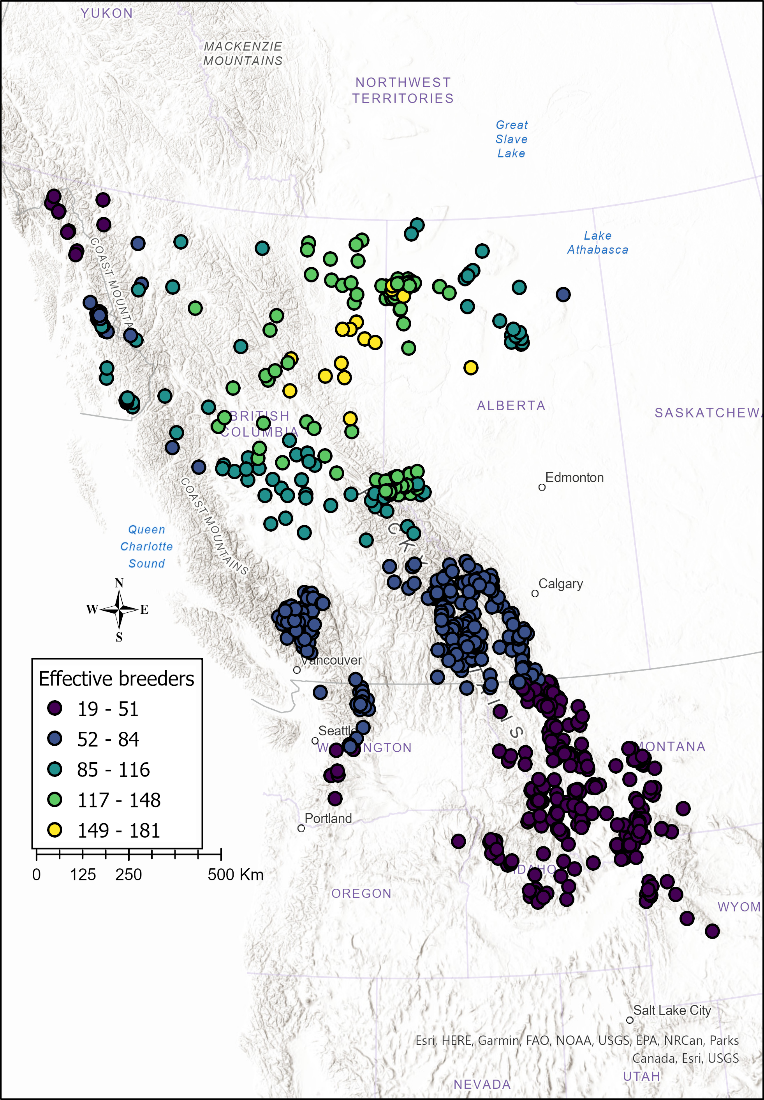

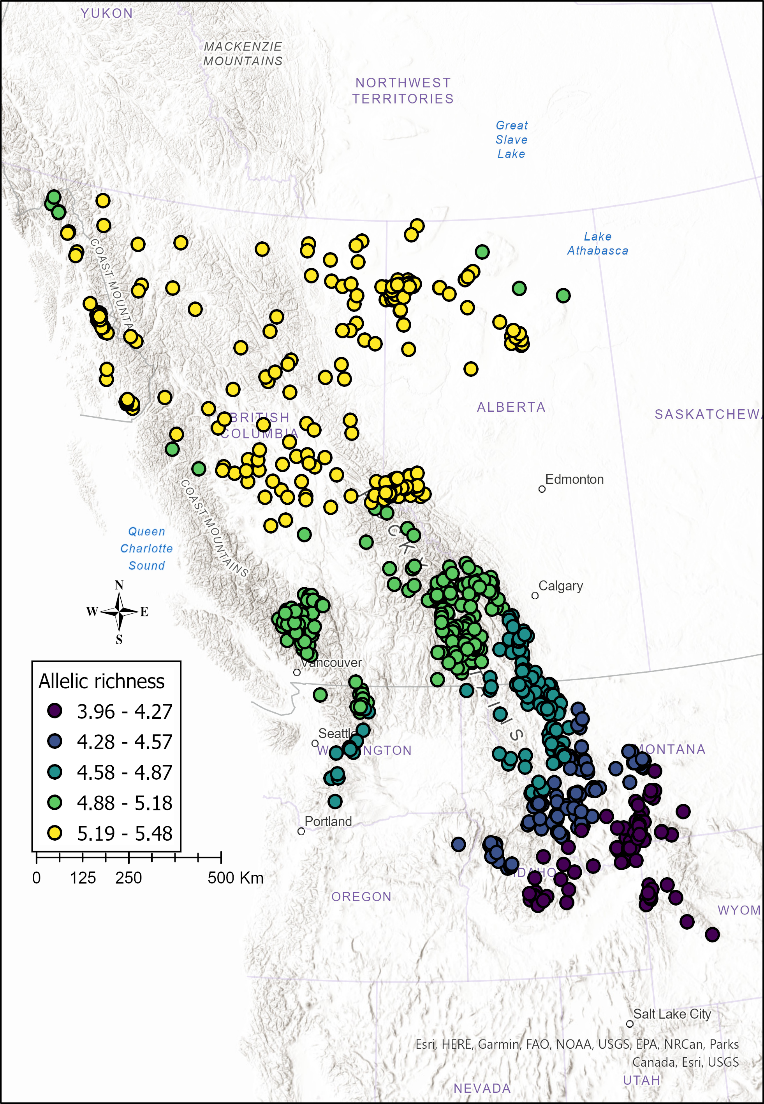


**(A)**

**(B)**

**(C)**

**(D)**

**Supplementary Figure 3: Spatial genetic diversity.** Within the spatial-genetic neighborhood (555 km), (A) the number of individuals used to calculate estimates within the radius for (B) allelic richness across all loci/individuals, (C) average inbreeding coefficient (Fixation Index) across all loci/individuals, and (D) effective number of breeding individuals (Wright's neighborhood size), excluding rare alleles with a frequency of less than 0.10 that could bias the estimate.


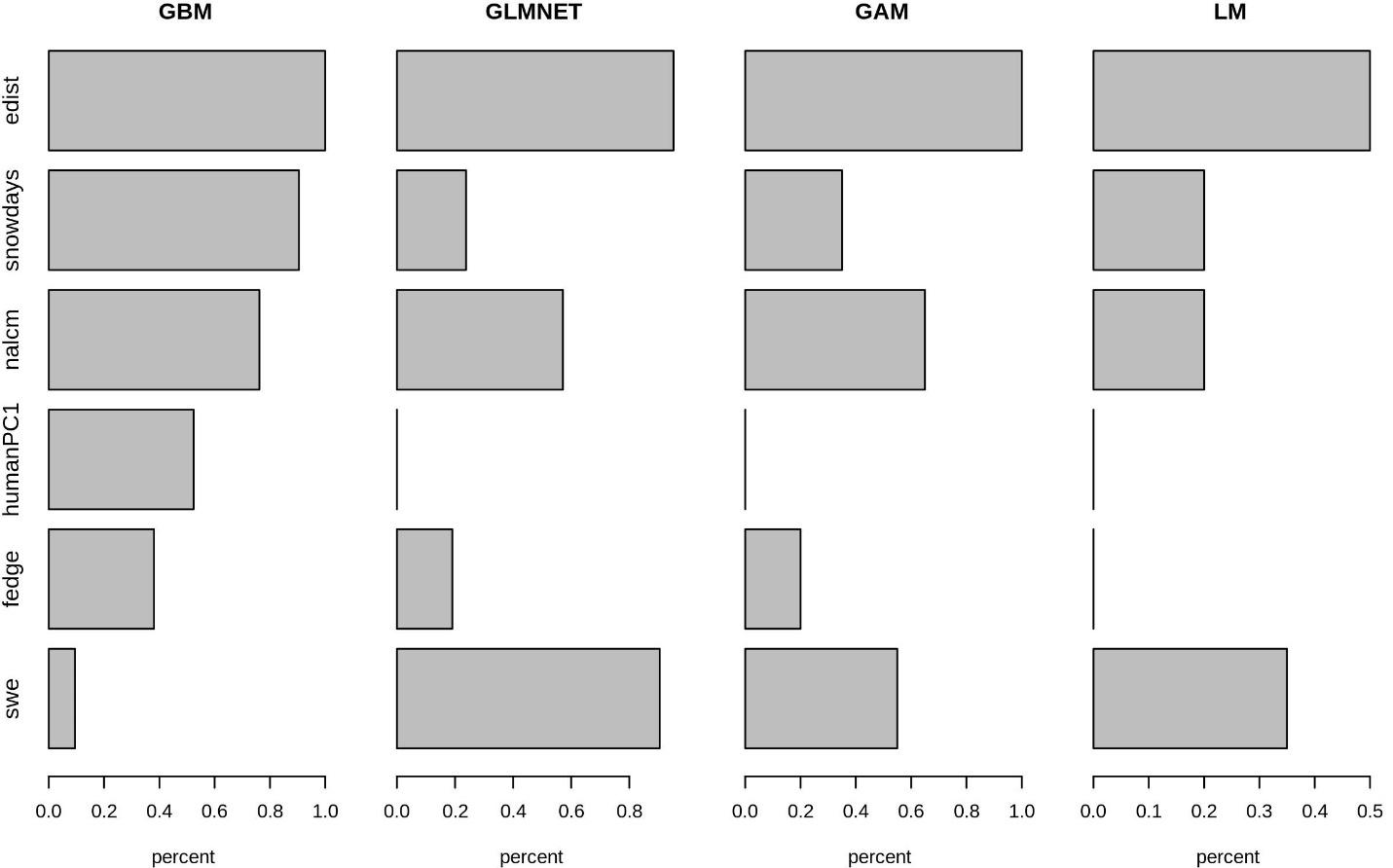


**Supplementary Figure 4: Least cost transect analysis (LCTA) variable selection.** Variable selection results from the feature selection step of the LCTA. The X-axis represents the percentage of models that a given variable was retained. Random Forests is not shown here and selected all variables 100% of the time. A final GBM model with and without human disturbance PC1 was tested for model accuracy: 3 variable model (geographic distance, snow days, forest cover) RMSE = 0.07698, *R^2^* = 0.1936, and MAE = 0.0609 and 4 variables (geographic distance, snow days, forest cover and human disturbance PC1) RMSE = 0.07675, *R^2^* = 0.1974, and MAE = 0.0604. GBM – gradient boosting machine, GLMNET – generalized linear model elastic-net regularized, RF – random forests, GAM – generalized additive model, LM – linear model. Edist – Euclidean distance, nalcm – forest cover, humanPC1 – human disturbance, fedge – forest edge, swe – snow-water equivalent.

**
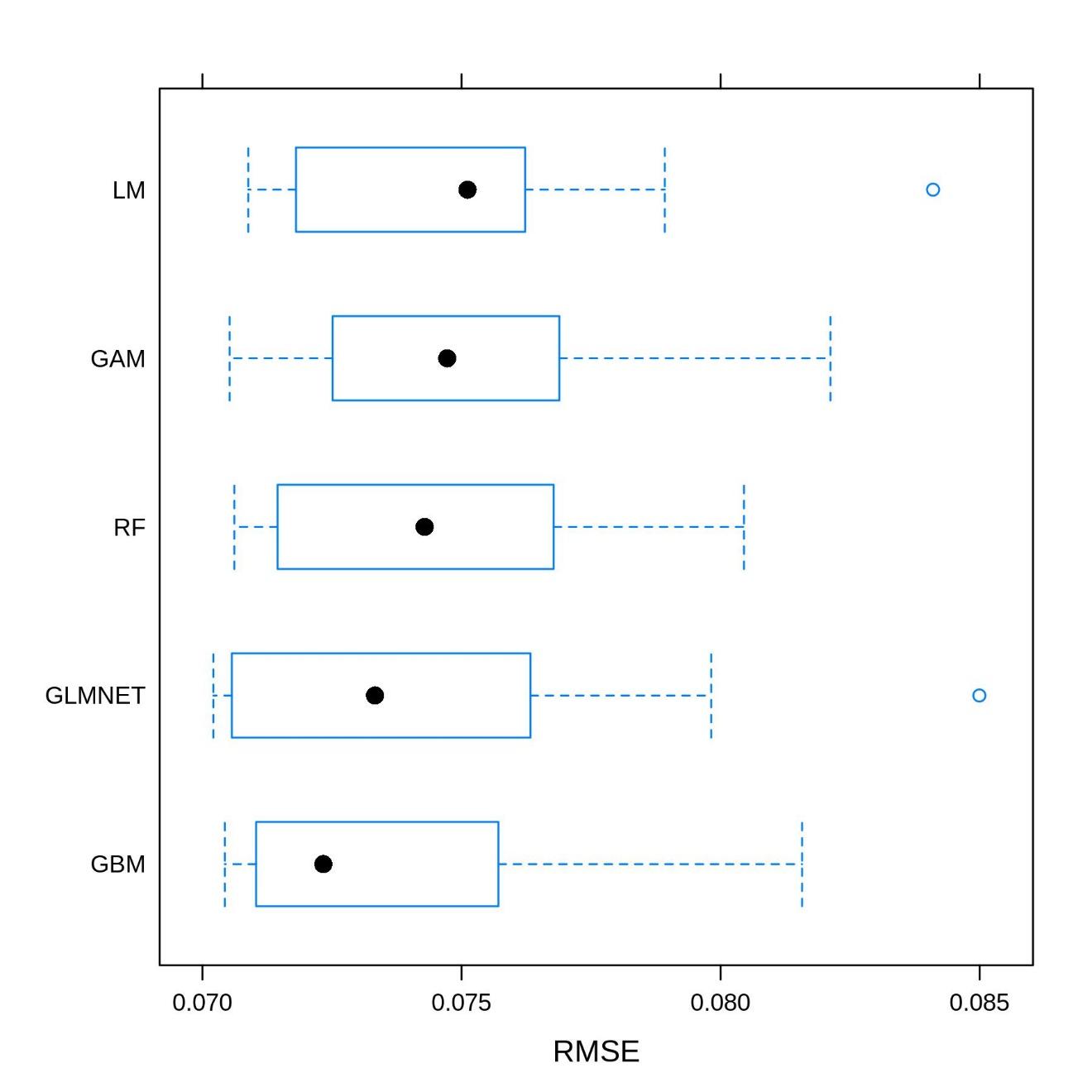
**

**Supplementary Figure 5: Least cost transect analysis (LCTA) feature selection models.** Root mean squared error (RMSE) results for the 5 different variable selection models applied to the LCTA iterative output. GBM – gradient boosting machine, GLMNET – generalized linear model elastic-net regularized, RF – random forests, GAM – generalized additive model, LM – linear model.

**
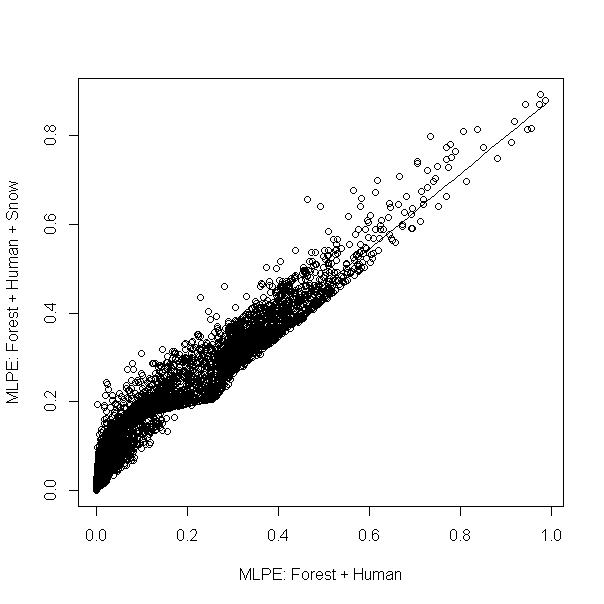
**

**Supplementary Figure 6: MLPE prediction surfaces compared.** 5,000 random raster grid cells and their corresponding relationship for the top two MLPE prediction surfaces (forest cover and human disturbance; x-axis and forest cover, human disturbance, and snow; y-axis). Pearson’s correlation, *r* = 0.964.


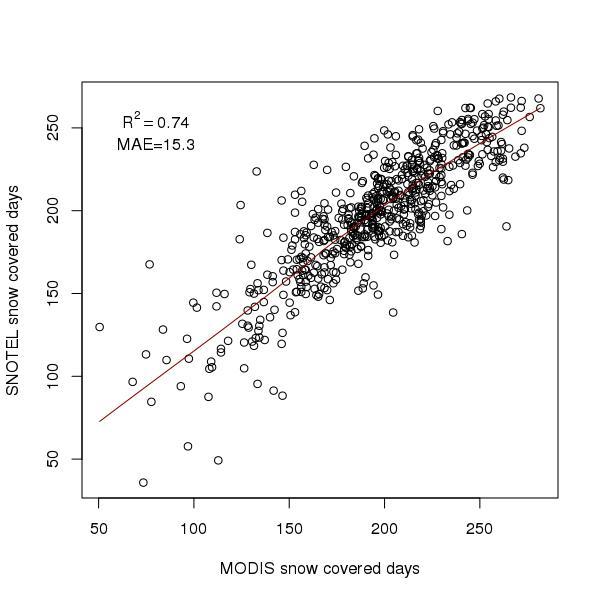


**Supplementary Figure 7: Validation of the snow days layer against United States SNOTEL station data.** This layer represents the total number of 8-day periods with snow cover from 2002-2018. The scale (250 m) is finer than other available snow products and able to better capture aspect effects on snow cover. The layer was produced by counting the NoData pixels (surrogate for snow) in the USGS NDVI layer (see Table 2) across the 17-year period. These data were then validated against US Snotel station data.


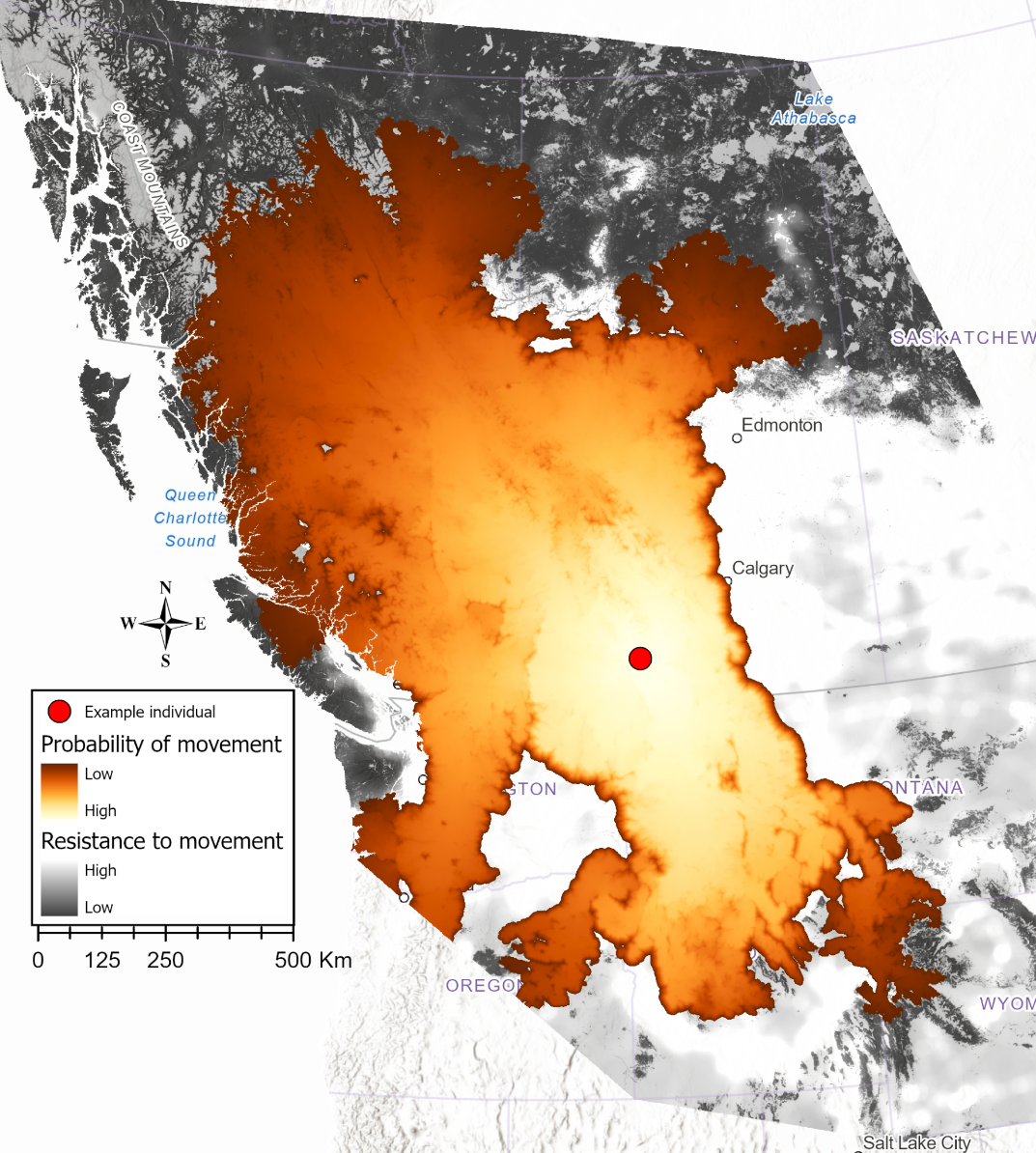


**Supplementary Figure 8: Effective distance kernel.** Example point in center of study area with a 30 percent maximum effective distance threshold applied using the landscape resistance surface (white - high resistance areas; black - low resistance areas). Light yellow areas reflect higher movement probability areas and dark brown shows the extent of this individual's movement distribution.


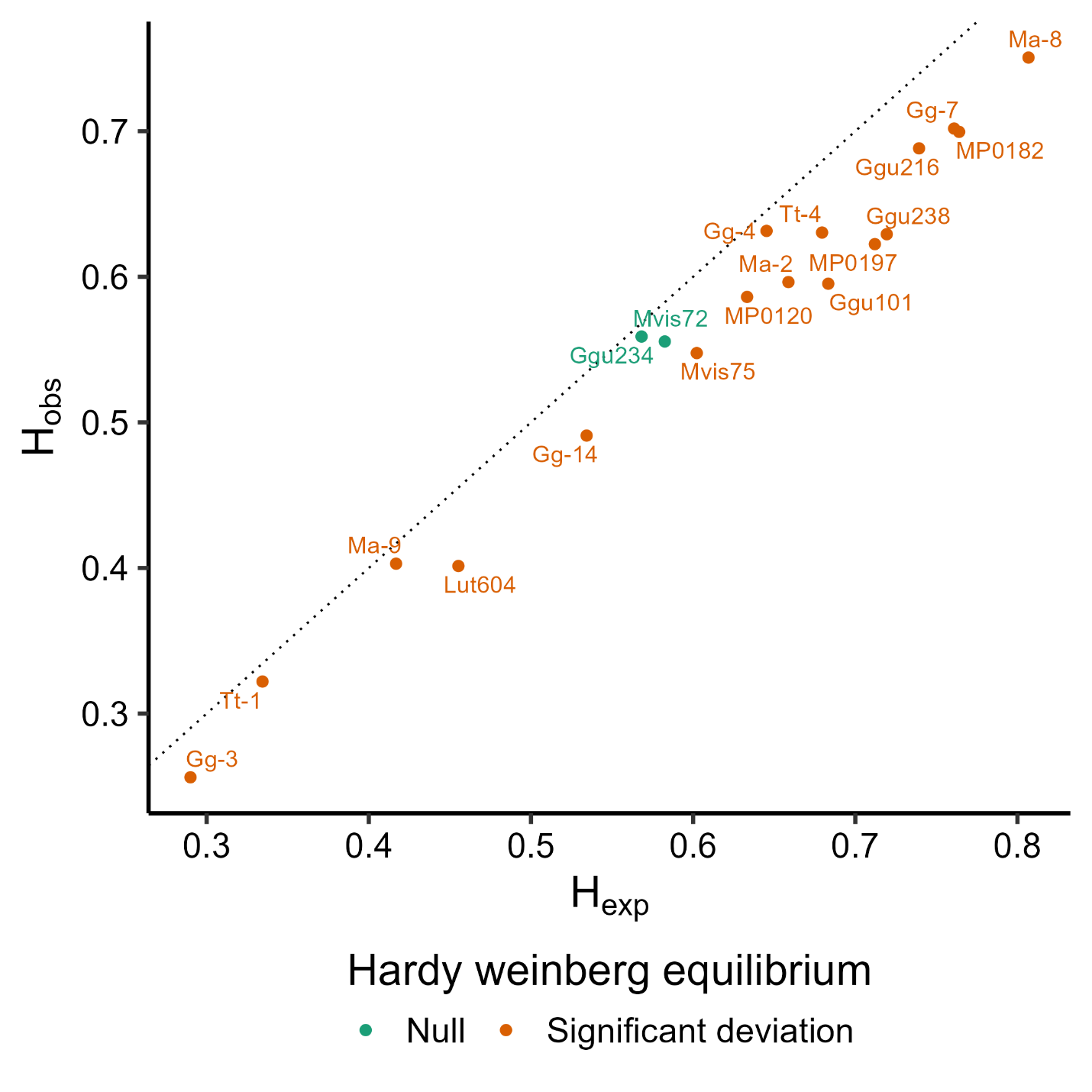
**Supplementary Figure 9: Effective distance kernel.** Exa

**Supplementary Tables 1 – 5**

**Supplementary Table 1: Model selection for scale tests.** Two-step hierarchical model selection results for maximum likelihood population effects mixed model regression of wolverine genetic distances and landscape resistance based on least cost paths. Fine and broad scale patterns refer to the samples that occur at a scale less than 555 km and samples greater than 555 km, respectively. Step one evaluated only univariate models and selected variables for the multi-variable step based on whether they outperformed distance alone. For each variable, four scales (1, 10, 100, 1,000 km^2^) and three transformations (linear, upper exponential, and lower exponential) were tested, and the best performing variable is presented. Variables for the multi-variable step were produced by taking a weighted average across variable rasters and recalculating least cost paths. Distance and log of distance represent the null model of isolation-by-distance. AIC is used for ranking MLPE models across both univariate and multi-variable models, and not for evaluating relative performance among models. SWE = snow water equivalent; TRI = topographic ruggedness index; TPI = topographic position index; PC1 = principal component (axis 1). The best performing models are in bold font.

| **Variables** | **Scale (km^2^)** | **Transformation** | **marg. R^2^** | **cond. R^2^** | **Δ AICc** |
| --- | --- | --- | --- | --- | --- |
| **Broad scale - Univariate** |  |  |  |  |  |
| Climate PC1 | 1000 | linear | 0.049 | 0.186 | 288.0 |
| Snow days | 100 | linear | 0.066 | 0.210 | 315.7 |
| Forest edge | 10 | lower | 0.050 | 0.179 | 319.3 |
| Human footprint 2 | 1000 | lower | 0.044 | 0.178 | 340.6 |
| Human footprint 1 | 100 | lower | 0.043 | 0.175 | 385.0 |
| Distance | NA | NA | 0.042 | 0.172 | 436.5 |
| log of Distance | NA | NA | 0.037 | 0.166 | 452.4 |
| SWE | 100 | linear | 0.042 | 0.175 | 477.9 |
| Forest cover | 1 | lower | 0.056 | 0.189 | 480.1 |
| Human disturbance PC1 | 1000 | upper | 0.166 | 0.372 | 551.6 |
| Elevation | 1 | upper | 0.040 | 0.171 | 606.3 |
| TRI | 10 | upper | 0.040 | 0.171 | 607.9 |
| Building density | 1000 | lower | 0.040 | 0.171 | 612.4 |
| NASA Lights | 1000 | lower | 0.040 | 0.171 | 619.2 |
| TPI | 1 | upper | 0.040 | 0.171 | 619.9 |
| Highway 1 | NA | NA | 0.016 | 0.146 | 1402.1 |
| All highways | NA | NA | 0.020 | 0.154 | 2130.8 |
| **Broad scale - Multivariate** |  |  |  |  |  |
| **Forest edge + Human footprint 2** | |  | **0.051** | **0.182** | **0.0** |
| Climate PC1 + Human footprint 2 | |  | 0.051 | 0.189 | 251.2 |
| Climate PC1 + Forest edge + Human footprint 2 | | | 0.047 | 0.182 | 324.6 |
| Climate PC1 + Forest edge |  |  | 0.046 | 0.180 | 337.5 |

**Supplementary Table 1 continued**

| **Variables** | **Scale (sq km)** | **Transformation** | **marg. R^2^** | **cond. R^2^** | **Δ AICc** |
| --- | --- | --- | --- | --- | --- |
| **Fine scale - Univariate** |  |  |  |  |  |
| Forest cover | 10 | lower | 0.101 | 0.266 | 22.9 |
| Human footprint 2 | 1000 | lower | 0.064 | 0.200 | 994.0 |
| log of Distance | NA | NA | 0.060 | 0.193 | 1006.4 |
| Human disturbance PC1 | 1000 | lower | 0.061 | 0.193 | 1404.4 |
| Forest edge | 1000 | linear | 0.064 | 0.196 | 1404.5 |
| Building density | 1000 | lower | 0.060 | 0.192 | 1510.3 |
| NASA lights | 1000 | lower | 0.060 | 0.192 | 1515.0 |
| Human footprint 1 | 10 | lower | 0.061 | 0.194 | 1517.7 |
| TRI | 1 | upper | 0.060 | 0.192 | 1535.5 |
| TPI | 100 | upper | 0.060 | 0.192 | 1548.8 |
| Climate PC1 | 100 | upper | 0.060 | 0.192 | 1559.7 |
| Distance | NA | NA | 0.060 | 0.191 | 1568.2 |
| SWE | 100 | upper | 0.060 | 0.192 | 1576.3 |
| Elevation | 1 | upper | 0.061 | 0.190 | 1579.6 |
| Snow days | 100 | upper | 0.060 | 0.195 | 1607.2 |
| All highways | NA | NA | 0.032 | 0.173 | 7137.1 |
| Highway 1 | NA | NA | 0.006 | 0.139 | 10397.0 |
| **Fine scale - Multivariate** |  |  |  |  |  |
| **Forest cover + Human footprint 2** | |  | **0.087** | **0.244** | **0** |

**Supplementary Table 2: Male and female population tests.** Two-step hierarchical model selection results for maximum likelihood population effects mixed model regression of sex-specific wolverine genetic distances and landscape resistance based on least cost paths. Step one evaluated only univariate models and selected variables for the multi-variable step based on whether they outperformed distance alone. For each variable, four scales (1, 10, 100, 1,000 km^2^) and three transformations (linear, upper exponential, and lower exponential) were tested, and the best performing variable is presented. Variables for the multi-variable step were produced by taking a weighted average across variable rasters and recalculating least cost paths. Distance and log of distance represent the null model of isolation-by-distance. AIC is used for ranking MLPE models across both univariate and multi-variable models, and not for evaluating relative performance among models. SWE = snow water equivalent; TRI = topographic ruggedness index; TPI = topographic position index; PC1 = principal component (axis 1). For the sake of brevity, we only include the top 6 multi-variable models. The best performing models are in bold font.

| **Variables** | **Scale (km^2^)** | **Transformation** | **marg. R^2^** | **cond. R^2^** | **Δ AICc** |
| --- | --- | --- | --- | --- | --- |
| **Female - Univariate** |  |  |  |  |  |
| **Log of distance** | **NA** | **NA** | **0.147** | **0.247** | **0** |
| Human disturbance PC1 | 1000 | Upper | 0.175 | 0.339 | 582 |
| Human footprint 2 | 1000 | Upper | 0.198 | 0.375 | 808 |
| Forest Cover | 1 | Linear | 0.194 | 0.319 | 854 |
| Snow days | 1000 | Lower | 0.181 | 0.314 | 895 |
| Climate PC1 | 1000 | Lower | 0.164 | 0.293 | 1010 |
| SWE | 1000 | Lower | 0.157 | 0.275 | 1385 |
| Human footprint 1 | 1000 | Lower | 0.152 | 0.262 | 1596 |
| TRI | 1000 | Upper | 0.158 | 0.266 | 1905 |
| Building density | 1000 | Lower | 0.152 | 0.260 | 1988 |
| Distance | NA | NA | 0.153 | 0.261 | 2008 |
| Lights | 1000 | Lower | 0.152 | 0.260 | 2008 |
| TPI | 10 | Upper | 0.152 | 0.261 | 2028 |
| Forest edge | 1 | Upper | 0.152 | 0.260 | 2033 |
| Elevation | 1 | Upper | 0.155 | 0.262 | 2050 |
| Highways all | NA | NA | 0.095 | 0.213 | 3552 |
| Highways 1 | NA | NA | 0.048 | 0.147 | 7036 |
| **Female - Multivariate** |  |  |  |  |  |
| Forest Cover + Human disturbance PC1 | |  | 0.178 | 0.323 | 7 |
| Forest Cover + Human disturbance PC1 + Snow days | | | 0.179 | 0.318 | 57 |
| Human disturbance PC1 + Snow days | | | 0.174 | 0.321 | 356 |
| Forest Cover + Snow days |  |  | 0.193 | 0.324 | 551 |

**Supplementary Table 2 continued**

| **Variables** | **Scale (km^2^)** | **Transformation** | **marg. R^2^** | **cond. R^2^** | **Δ AICc** |
| --- | --- | --- | --- | --- | --- |
| **Male - Univariate** |  |  |  |  |  |
| Snow days | 1000 | Linear | 0.146 | 0.266 | 707 |
| Climate PC1 | 100 | Lower | 0.142 | 0.261 | 793 |
| Human footprint 2 | 1000 | Lower | 0.129 | 0.237 | 1064 |
| Forest cover | 1 | Linear | 0.149 | 0.262 | 1123 |
| Human disturbance PC1 | 1000 | Linear | 0.166 | 0.328 | 1138 |
| Human footprint 1 | 1000 | Lower | 0.127 | 0.234 | 1207 |
| SWE | 1000 | Linear | 0.123 | 0.228 | 1593 |
| Distance | NA | NA | 0.127 | 0.232 | 1606 |
| Building density | 1000 | Lower | 0.126 | 0.231 | 1652 |
| Elevation | 1 | Upper | 0.128 | 0.231 | 1668 |
| Lights | 1000 | Lower | 0.126 | 0.231 | 1673 |
| TRI | 10 | Upper | 0.126 | 0.231 | 1682 |
| TPI | 10 | Upper | 0.126 | 0.231 | 1688 |
| Forest edge | 10 | Upper | 0.126 | 0.231 | 1689 |
| Log of Distance | NA | NA | 0.103 | 0.200 | 2640 |
| Highways all | NA | NA | 0.087 | 0.207 | 4976 |
| Highway 1 | NA | NA | 0.064 | 0.167 | 6722 |
| **Male - Multivariate** |  |  |  |  |  |
| **Human footprint 2 + Forest cover** | | | **0.149** | **0.285** | **0** |
| Human footprint 2 + Forest cover + Snow days | | | 0.144 | 0.263 | 415 |
| Human footprint 2 + Snow days | | | 0.148 | 0.272 | 539 |
| Forest cover + Snow days | | | 0.144 | 0.259 | 589 |

**Supplementary Table 3: Northern and southern population tests.** Two-step hierarchical model selection results for maximum likelihood population effects mixed model regression of wolverine genetic distances and landscape resistance based on least cost paths. Northern and southern populations refer to the samples that occur north and south of Highway 1 in Canada, respectively. Step one evaluated only univariate models and selected variables for the multi-variable step based on whether they outperformed distance alone. For each variable, four scales (1, 10, 100, 1,000 km^2^) and three transformations (linear, upper exponential, and lower exponential) were tested, and the best performing variable is presented. Variables for the multi-variable step were produced by taking a weighted average across variable rasters and recalculating least cost paths. Distance and log of distance represent the null model of isolation-by-distance. AIC is used for ranking MLPE models across both univariate and multi-variable models. SWE = snow water equivalent; TRI = topographic ruggedness index; TPI = topographic position index; PC1 = principal component (axis 1). For brevity, we only include the top 6 multi-variable models. The best performing models are in bold font. No multi-variable models were tested for the northern population, since only one variable outperformed distance.

| **Variables** | **Scale (sq km)** | **Transformation** | **marg. R^2^** | **cond. R^2^** | **Δ AICc** |
| --- | --- | --- | --- | --- | --- |
| **Northern population -Univariate** | |  |  |  |  |
| **Forest cover** | **10** | **linear** | **0.095** | **0.189** | **0** |
| log of Distance | NA | NA | 0.066 | 0.155 | 240.9 |
| Snow days | 100 | linear | 0.074 | 0.164 | 392.2 |
| Climate PC1 | 1000 | linear | 0.068 | 0.157 | 499.2 |
| Elevation | 100 | linear | 0.075 | 0.165 | 499.3 |
| Human footprint 1 | 1000 | lower | 0.070 | 0.161 | 553.4 |
| TPI | 10 | lower | 0.070 | 0.160 | 566.2 |
| TRI | 1 | lower | 0.070 | 0.160 | 572.1 |
| Human footprint 2 | 1000 | lower | 0.073 | 0.164 | 585.5 |
| Human disturbance PC1 | 1000 | lower | 0.073 | 0.164 | 645.6 |
| SWE | 100 | upper | 0.071 | 0.162 | 661.0 |
| Building density | 1000 | lower | 0.073 | 0.164 | 673.8 |
| NASA lights | 1000 | lower | 0.073 | 0.164 | 675.2 |
| Distance | NA | NA | 0.073 | 0.164 | 679.7 |
| Forest edge | 1 | upper | 0.073 | 0.164 | 685.2 |
| All highways | NA | NA | 0.020 | 0.112 | 4481.1 |
| Highway 1 | NA | NA | 0.001 | 0.089 | 5887.5 |
| **Southern population - Univariate** | |  |  |  |  |
| **Forest cover** | **1** | **lower** | **0.218** | **0.412** | **0** |
| TRI | 100 | lower | 0.152 | 0.332 | 1686.6 |
| Human disturbance PC1 | 1000 | linear | 0.150 | 0.323 | 1786.1 |
| Human footprint 2 | 1000 | upper | 0.152 | 0.335 | 1925.1 |
| Forest edge | 100 | lower | 0.121 | 0.276 | 2384.0 |
| Snow days | 10 | lower | 0.126 | 0.293 | 2390.1 |
| log of Distance | NA | NA | 0.093 | 0.248 | 2774.8 |
| Climate PC1 | 1 | lower | 0.112 | 0.276 | 2935.7 |
| **Supplementary Table 3 continued** | | | | | |
| SWE | 1000 | lower | 0.105 | 0.264 | 2988.0 |
| Human footprint 1 | 1000 | lower | 0.114 | 0.268 | 3010.7 |
| Elevation | 1 | lower | 0.141 | 0.282 | 3238.1 |
| Building density | 1000 | lower | 0.103 | 0.256 | 3490.1 |
| TPI | 10 | upper | 0.103 | 0.257 | 3492.4 |
| Distance | NA | NA | 0.103 | 0.256 | 3492.5 |
| NASA lights | 1000 | lower | 0.103 | 0.256 | 3494.6 |
| All highways | NA | NA | 0.056 | 0.220 | 8021.8 |
| Highway 1 | NA | NA | 0.003 | 0.162 | 14287.1 |
| **Southern population - Multivariate** | |  |  |  |  |
| Forest cover + Human disturbance PC1 | |  | 0.199 | 0.386 | 203.0 |
| Forest cover + Snow days | |  | 0.175 | 0.359 | 460.9 |
| Forest cover + Human disturbance PC1 + Snow days | | | 0.174 | 0.359 | 501.6 |
| Forest cover + TRI |  |  | 0.175 | 0.362 | 591.0 |
| Forest cover + TRI + Snow days | |  | 0.169 | 0.352 | 665.5 |
| Forest cover + TRI + Human disturbance | | | 0.172 | 0.358 | 680.1 |

**Supplementary Table 4: Multicollinearity between all variables tested.** Spatial correlations among the raw raster data for all variables following the linear conversion to resistance. TRI = topographic ruggedness index; TPI = topographic position index; PC = principal component; HF = Human footprint; SWE = Snow water equivalent

|  | **Buildings** | **Climate PC** | **Elevation** | **Forest edge** | **HF 2** | **HF**  **PC** | **HF 1** | **Lights** | **Forest cover** | **Snow days** | **SWE** | **Temp- erature** | **TPI** | **TRI** |
| --- | --- | --- | --- | --- | --- | --- | --- | --- | --- | --- | --- | --- | --- | --- |
| **Buildings** | 1.00 | 0.08 | 0.07 | 0.05 | 0.31 | 0.81 | 0.45 | 0.80 | 0.07 | 0.07 | 0.03 | 0.06 | 0.00 | 0.05 |
| **Climate PC** | 0.08 | 1.00 | 0.33 | 0.23 | 0.37 | 0.25 | 0.33 | 0.08 | 0.33 | 0.92 | 0.65 | 0.89 | 0.09 | 0.50 |
| **Elevation** | 0.07 | 0.33 | 1.00 | 0.02 | 0.23 | 0.14 | 0.09 | 0.07 | -0.10 | 0.25 | 0.10 | 0.11 | 0.11 | 0.35 |
| **Forest edge** | 0.05 | 0.23 | 0.02 | 1.00 | 0.30 | 0.18 | 0.20 | 0.06 | 0.36 | 0.28 | 0.02 | 0.27 | -0.01 | 0.19 |
| **Human Footprint 2** | 0.31 | 0.37 | 0.23 | 0.30 | 1.00 | 0.67 | 0.60 | 0.35 | 0.40 | 0.36 | 0.16 | 0.33 | 0.01 | 0.26 |
| **Human footprint PC** | 0.81 | 0.25 | 0.14 | 0.18 | 0.67 | 1.00 | 0.82 | 0.86 | 0.25 | 0.25 | 0.10 | 0.24 | 0.01 | 0.15 |
| **Human footprint 1** | 0.45 | 0.33 | 0.09 | 0.20 | 0.60 | 0.82 | 1.00 | 0.55 | 0.29 | 0.33 | 0.13 | 0.34 | 0.02 | 0.16 |
| **Lights** | 0.80 | 0.08 | 0.07 | 0.06 | 0.35 | 0.86 | 0.55 | 1.00 | 0.08 | 0.08 | 0.03 | 0.06 | 0.00 | 0.05 |
| **Forest cover** | 0.07 | 0.33 | -0.10 | 0.36 | 0.40 | 0.25 | 0.29 | 0.08 | 1.00 | 0.38 | 0.02 | 0.44 | -0.06 | 0.18 |
| **Snow days** | 0.07 | 0.92 | 0.25 | 0.28 | 0.36 | 0.25 | 0.33 | 0.08 | 0.38 | 1.00 | 0.41 | 0.82 | 0.06 | 0.42 |
| **SWE** | 0.03 | 0.65 | 0.10 | 0.02 | 0.16 | 0.10 | 0.13 | 0.03 | 0.02 | 0.41 | 1.00 | 0.40 | 0.07 | 0.38 |
| **Temperature** | 0.06 | 0.89 | 0.11 | 0.27 | 0.33 | 0.24 | 0.34 | 0.06 | 0.44 | 0.82 | 0.40 | 1.00 | 0.06 | 0.37 |
| **TPI** | 0.00 | 0.09 | 0.11 | -0.01 | 0.01 | 0.01 | 0.02 | 0.00 | -0.06 | 0.06 | 0.07 | 0.06 | 1.00 | 0.11 |
| **TRI** | 0.05 | 0.50 | 0.35 | 0.19 | 0.26 | 0.15 | 0.16 | 0.05 | 0.18 | 0.42 | 0.38 | 0.37 | 0.11 | 1.00 |

**Supplementary Table 5:** Descriptive statistics using ‘genalex’ package in R and by locus (N=19) and by mean across loci using 882 wolverines from the western United States and Canada. Note that He is expected to be positively biased due to population structure. The 19 loci include 16 loci described in Schwartz et al. (2009) (Gg3, Gg4, Gg7, Ma2, Ma8, Ma9, Tt1, Tt4, Ggu101, Ggu216, Ggu234, Ggu238, Mvis020, Mvis72, Mvis075, Lut604) and 3 loci described in Jordan et al. (2007). The results for Hardy Weinberg Equilibrium are shown in grey from the package ‘pegas’ and function hw.test() with 1000 Monte Carlo replicates.

| **Locus** | **N** | **A** | **Ho** | **uHe** | **chi^2** | **df** | **Pr(chi^2 >)** | **Pr.exact** |  |
| --- | --- | --- | --- | --- | --- | --- | --- | --- | --- |
| Ma-2 | 882 | 4.0 | 0.596 | 0.659 | 37.97215 | 6 | 1.14E-06 | 0 |  |
| Gg-4 | 882 | 6.0 | 0.632 | 0.646 | 32.94904 | 15 | 0.004771 | 0.01 |  |
| Gg-7 | 882 | 7.0 | 0.702 | 0.761 | 77.84073 | 21 | 1.85E-08 | 0 |  |
| Ggu101 | 882 | 7.0 | 0.595 | 0.684 | 117.7575 | 21 | 1.89E-15 | 0 |  |
| Ggu216 | 882 | 7.0 | 0.688 | 0.740 | 69.48248 | 21 | 4.25E-07 | 0 |  |
| Tt-4 | 882 | 4.0 | 0.630 | 0.680 | 16.40586 | 6 | 0.011734 | 0.011 |  |
| Mvis75 | 882 | 5.0 | 0.548 | 0.603 | 30.61612 | 10 | 6.79E-04 | 0.002 |  |
| Gg-14 | 882 | 8.0 | 0.491 | 0.535 | 184.4467 | 28 | 0 | 0.001 |  |
| Lut604 | 882 | 8.0 | 0.401 | 0.456 | 104.3923 | 28 | 9.72E-11 | 0 |  |
| MP0182 | 882 | 9.0 | 0.700 | 0.764 | 187.4624 | 36 | 0 | 0 |  |
| MP0197 | 882 | 7.0 | 0.622 | 0.712 | 109.4181 | 21 | 6.01E-14 | 0 |  |
| Gg-3 | 882 | 4.0 | 0.256 | 0.290 | 46.92805 | 6 | 1.93E-08 | 0 |  |
| Ma-9 | 881 | 4.0 | 0.403 | 0.417 | 1054.456 | 6 | 0 | 0 |  |
| MP0120 | 882 | 8.0 | 0.586 | 0.634 | 59.37279 | 28 | 4.89E-04 | 0.001 |  |
| Mvis72 | 882 | 4.0 | 0.556 | 0.583 | 7.806563 | 6 | 0.25262 | 0.099 |  |
| Tt-1 | 882 | 5.0 | 0.322 | 0.335 | 19.48371 | 10 | 0.034532 | 0.034 |  |
| Ggu234 | 882 | 5.0 | 0.559 | 0.569 | 13.51684 | 10 | 0.196192 | 0.115 |  |
| Ggu238 | 882 | 6.0 | 0.629 | 0.720 | 121.9643 | 15 | 0 | 0 |  |
| Ma-8 | 882 | 10.0 | 0.751 | 0.807 | 175.7504 | 45 | 0 | 0 |  |
| Mean | 881.9 | 6.2 | 0.561 | 0.610 |  |  |  |  |  |
| SE | 0.053 | 0.430 | 0.030 | 0.034 |  |  |  |  |  |
|  |  | | | | | | | | |
|  |  | | | | | | | | |

**Supplementary Data 1 – 3**These data sets together will allow for reproducibility of all statistical analyses performed in the paper.

**Supplementary Data 1.** Dataframe of genetic distances in columnar form (rows = 882 * 881 / 2) for all MLPE subsets (full, male, females, north, sourth, broad scale, finescale). See repository file link.

**Supplementary Data 2.** All univariate cost distance matrices used in the MLPE method. All other cost distance matrices can be derived from this using the columnar data to define which individuals to select from each subset. See repository file link.

**Supplementary Data 3**. A layer pack for ArcGIS that contains the visualizations presented in the main text Figure 2.
